# Human GV oocytes generated by mitotically active germ cells obtained from follicular aspirates

**DOI:** 10.1038/srep28218

**Published:** 2016-06-30

**Authors:** Xinbao Ding, Guishu Liu, Bo Xu, Changqing Wu, Ning Hui, Xin Ni, Jian Wang, Meirong Du, Xiaoming Teng, Ji Wu

**Affiliations:** 1 Renji Hospital Shanghai Jiaotong University School of Medicine, Key Laboratory for the Genetics of Developmental & Neuropsychiatric Disorders (Ministry of Education), Bio-X Institutes, Shanghai Jiao Tong University, Shanghai 200240, China; 2 The First People’s Hospital of Chenzhou, Chenzhou 42300, Hunan, China; 3 Changhai Hospital of Second Military Medical University, Shanghai 200433, China; 4 Department of Physiology, Second Military Medical University, 800 Xiangyin Road, Shanghai 200433, China; 5 Laboratory for Reproductive Immunology, Obstetrics and Gynecology Hospital, Fudan University, Shanghai 200011, China.; 6 Shanghai Key Laboratory of Female Reproductive Endocrine Related Diseases, Obstetrics and Gynecology Hospital, Fudan University, Shanghai, 200011, China; 7 Center of Reproductive medicine, Shanghai First Maternity and Infant Hospital, Tongji University School of Medicine, Shanghai 200040, China; 8 Key Laboratory of Fertility Preservation and Maintenance of Ministry of Education, Ningxia Medical University, Yinchuan 750004, China; 9 Shanghai Key Laboratory of Reproductive Medicine, Shanghai 200025, China

## Abstract

Human female germline stem cells (FGSCs) have opened new opportunities for understanding human oogenesis, delaying menopause, treating infertility, and providing a new strategy for preserving fertility. However, the shortage of adult human ovaries tissues available impedes their future investigations and clinical applications. Here, we have established FGSC lines from scarce ovarian cortical tissues that exist in follicular aspirates (faFGSCs), which are produced and discarded in *in vitro* fertilization centers worldwide. The faFGSCs have characteristics of germline stem cells involved in the gene expression profile, growth characteristics, and a normal karyotype consistent with that of FGSCs obtained from ovarian cortexes surgically removed from patients (srFGSCs). Furthermore, faFGSCs have developmental potentials including spontaneous differentiation into oocytes under feeder-free conditions, communicating with granulosa cells by gap junctions and paracrine factors, entering meiosis after RA induction, as well as forming follicles after injection into human ovarian cortical tissues xenografted into adult immunodeficient female mice. Lastly, we developed a strategy guiding FGSCs differentiated into germinal vesicle (GV) stage oocytes *in vitro* and revealed their developmental mechanisms. Our study not only provides a new approach to obtain human FGSCs for medical treatment, but also opens several avenues to investigate human oogenesis *in vitro*.

Infertility affects ~48.5 million couples worldwide^1^ and becomes a major health and social concern. Germline stem cells (GSCs) are proposed for investigating the precise molecular mechanisms of infertility. In male mammals, spermatogonial stem cells (SSCs) maintain spermatogenesis via self-renewal and generation of spermatogonia destined to differentiate into spermatozoa^2^. By contrast, in most female mammals, it has been generally accepted that the number of oocytes available throughout life is determined during the perinatal period^3^,^4^,^5^,^6^. Johnson *et al*., however, suggested that there is ovarian regenerative activity in juvenile and adult mice by estimating follicle numbers and death (atresia) rates in mouse ovaries^7^. Subsequently, Johnson *et al*. identified bone marrow as a potential source of germ cells that could sustain ovarian regenerative activity in adulthood^8^. However, Eggan *et al*. indicated that bone marrow is not involved in the formation of mature ovulated oocytes^9^. We have previously found that FGSCs with the capacity to produce fully functional oocytes and fertile offspring after transplantation into ovaries could be successfully isolated and cultured from neonatal and adult mouse ovaries by magnetic-activated cell sorting (MACS) with the antibody against the C-terminal of DDX4 (DEAD box polypeptide 4)^10^,^11^. Additionally, using FGSCs we generated transgenic or gene knockdown mice^12^. Oogonial stem cells (OSCs, also termed FGSCs) with oogenic capacity were also successfully isolated and purified from adult mice and healthy reproductive-age women using fluorescence-activated cell sorting (FACS) with the same antibody^13^. We also showed similar morphological and molecular signatures shared by FGSCs and SSCs^14^. A growing body of research demonstrated that FGSCs could be achieved from mammalian ovary including mouse, rat, and human^11^,^12^,^13^,^14^,^15^,^16^,^17^,^18^,^19^,^20^,^21^,^22^,^23^,^24^,^25^,^26^. In particular, clinical trials performed in 2 independent IVF centers reported that AUGMENT^SM^ treatment (by means of injection of mitochondria from FGSCs into the patient’s own oocyte at the time of intracytoplasmic sperm injection) can improve embryo quality and increase the success of IVF^23^. However, different results for the existence of FGSCs were also reported^27^,^28^,^29^. For example, Zhang *et al*. claimed that Ddx4-expressing cells from postnatal mouse ovaries were not mitotically active using *Rosa26*^*rbw*/+^; *Ddx4-Cre* fluorescent reporter mice^29^. Countering this report, Park *et al*. found that the Cre-mediated recombination in *Rosa26; Ddx4-Cre* fluorescent reporter mice was “leaky”, and pointed out that Zhang *et al*. did not follow the protocols as described previously for isolating FGSCs^16^. In addition, Lei *et al*. claimed that adult mice ovaries lack GSCs using lineage tracing approach^28^ but they did not trace the development of individual germ cells.

Human FGSCs have opened new opportunities for understanding human oogenesis, delaying menopause, treating infertility, and providing a new strategy for preserving fertility. However, these applications are hindered by the shortage of adult human ovarian cortical biopsies available. This shortage prompted us to seek alternative sources of ovarian cortical tissues. Follicular aspirates (FAs) are produced during transvaginal oocyte retrieval, a technique used in ART (assisted reproductive technology) performed in over 1,000,000 cycles each year worldwide (Supplementary Table S1) to collect oocytes from the ovaries of women. They are often discarded after retrieval of the mature oocytes, leading to a large amount of waste. A previous study reported that preantral follicles could be captured in FAs^30^. Under *in vitro* culture, the oocytes from these follicles could grow, suggesting a new source of oocytes^30^. These findings, together with the discoveries on FGSCs, lead us to hypothesize that small pieces of ovarian cortical tissues containing FGSCs could be captured from FAs.

In humans, oogonia begin meiosis at approximately embryonic week 9, and form primordial follicles enclosed by pre-granulosa at approximately week 20^31^. However, the molecular mechanisms that govern these events are poorly understood owing to human fetal embryos being excluded from investigations, and the lack of *in vitro* models for tracking the early stages of oogenesis. FGSCs have been proposed to be a useful model to study the mechanisms of oogenesis *in vitro*. Park *et al*. reported that Bmp4 promotes mouse OSC differentiation through Smad1/5/8 activation, and up-regulates meiosis-initiating genes including *Msx1/2* and *Stra8 in vitro*^20^. Zhou *et al*. differentiated rat FGSCs into GV oocytes *in vitro*^24^. However, the processes and mechanisms underlying FGSC differentiation are largely unknown. All-trans retinoic acid (RA) is crucial for meiotic initiation by inducing the expression of a meiotic gatekeeper gene, *STRA8* (stimulated by retinoic acid gene 8)^32^. Studies on mice revealed that oocyte growth and development depends upon interactions with granulosa cells (GCs). These interactions are mainly mediated by gap junctions and by paracrine factors such as SCF (stem cell factor)^33^. However, very little is known about how RA and GCs regulate the differentiation of human FGSCs.

In this study, we have developed a method to isolate and culture FGSCs from scarce ovarian cortical tissues that exist in FAs (hereinafter referred to as faFGSCs) after oocyte retrieval and evaluated their capacities including, differentiation into oocytes, communicating with GCs, entering meiosis, and forming follicles associated with ovarian somatic cells. Furthermore, RA and GCs could facilitate the growth and development of oocytes differentiated by FGSCs *in vitro*. We also developed a strategy for differentiating FGSCs into GV oocytes *in vitro* and revealed their developmental mechanisms. Our study not only provides a new approach to obtain human FGSCs for medical treatment, but also opens several avenues to investigate human oogenesis *in vitro*.

## Results

### Isolation and characterization of human FGSCs from reproductive-age ovarian cortex of patients

**Figure 1:**
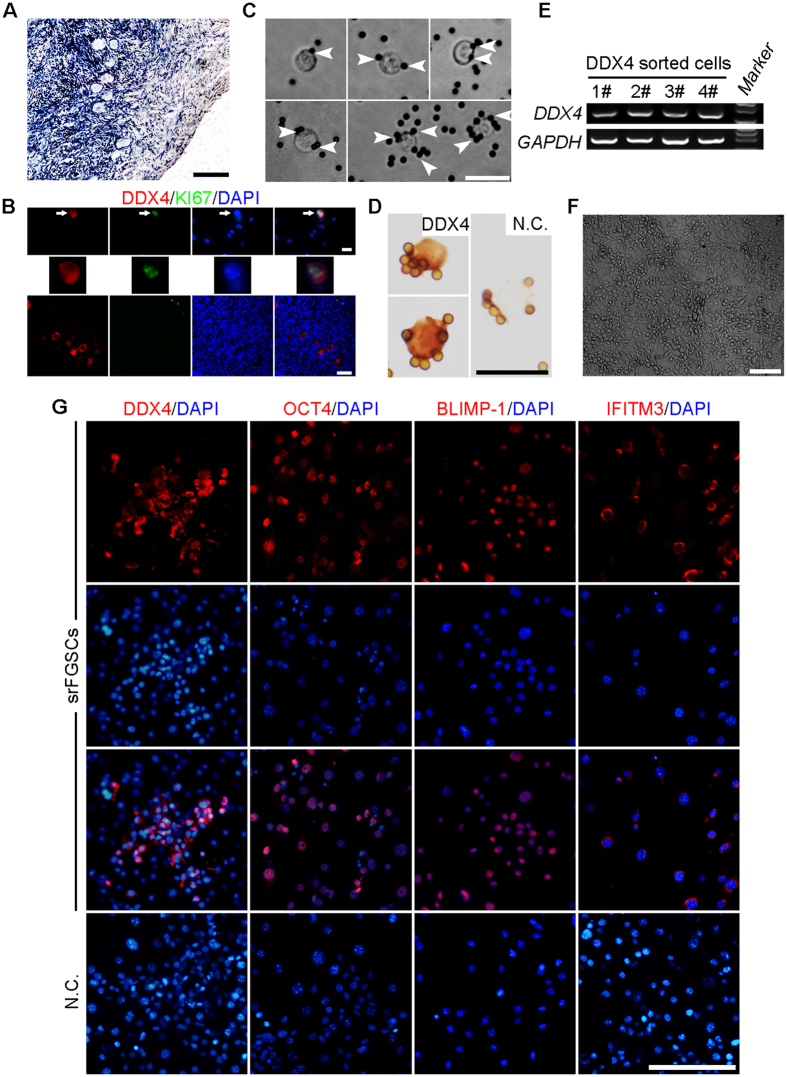
Isolation and characterization of human FGSCs from reproductive-age ovarian cortex of patients. (**A**) Representative histological appearance of adult human ovarian tissue used for the isolation of srFGSCs. (**B**) Representative images of DDX4 (red) and KI67 (green) in putative FGSCs (upper panel) and oocytes (lower panel) in human ovarian cortex. The image of putative FGSC (white arrows) is enlarged in middle panel. (**C**) Representative images of DDX4^+^ cells from reproductive-age ovarian cortex. White arrowheads indicate the magnetic beads. (**D**) Immunocytochemical analysis of DDX4 expression in srFGSCs using the antibody against the C-terminus of protein. (**E**) RT-PCR analysis of DDX4 sorted cells. Lane 5, 100-bp DNA markers. *GAPDH* is sample loading control. (**F**) A representative morphology of srFGSCs at passage 31. (**G**) SrFGSC line was detected by immunofluorescence analysis with the antibodies against DDX4, OCT4, IFITM3, and BLIMP-1. Negative control (N.C.) in (**D**,**G)** are the omission of the primary antibody. Full-length gels in E are presented in Supplementary Fig. 5. Scale bars: 10 μm (upper panel of **B**), 20 μm (**C**,**D**), 50 μm (**A**, lower panel of **B**,**G**), 100 μm (**F**).

We firstly isolated and cultured human srFGSCs from ovarian cortex biopsies surgically removed from patients with cervical carcinoma, ectopic pregnancy and ovarian cyst. The clinical data, procedure of sample taken and applications are summarized in Table 1. In line with previous studies^11^,^24^, we demonstrated that DDX4 and KI67 double-positive cells presented in human ovarian cortex, suggesting that they might be FGSCs (Fig. 1A,B). Using two-step enzymatic digestion and MACS of DDX4^+^ cells^11^,^12^,^13^, 50–150 round or ovoid cells with a diameter of 8–15 μm were obtained from patient ovarian tissues (n = 3, 26–43 years old) (Fig. 1C). The isolated cells had a morphology similar to freshly isolated DDX4^+^ cells from mouse ovaries^11^. Most of the cells had large cell bodies with little cytoplasm, slightly stained spherical nuclei. Furthermore, these isolated cells were confirmed as germ cells by immunocytochemical and reverse transcription polymerase chain reaction (RT-PCR) analysis (Fig. 1D,E). For culturing srFGSCs, DDX4^+^ cells were cultured in wells pre-seeded with inactive STO cells. At the initial passage, the candidate srFGSCs were subcultured every 5–7 days at a 1:1 ratio. As culturing continued, the number of srFGSCs gradually increased. After approximately 10 weeks in culture, proliferation of the cells become rapid and stable and subcultured every 5–6 days with a split ratio of 1:2. The srFGSCs had been passaged more than 30 times with a characteristic morphology similar to that of mouse FGSCs (Fig. 1F). Moreover, srFGSCs were used successfully to re-establish cell culture after cryopreservation and thawing. To characterize the srFGSCs, we determined the expression of *DDX4*, *IFITM3* (interferon-induced transmembrane protein 3), *OCT4* (also referred to as *POU5f*, POU-domain class-5 transcription factor), *STELLA* (also referred to as *DPPA3*, developmental pluripotency-associated 3), *DAZL* (deleted in azoospermia-like), *BLIMP-1* (also referred to as *PRDM1*, PR domain containing 1 with ZNF domain), *STRA8*, *SYCP3* (synaptonemal complex protein 3), *C-KIT* (also referred to as *SCFR*, stem cell factor receptor), *FIGLA* (factor in the germline alpha), *GDF9* (growth differentiation factor 9), *GJA4* (gap junction protein, alpha 4), *ZP1-3* (zona pellucida glycoprotein 1, 2, 3), *NANOG* (a pluripotency sustaining factor), *SOX-2* (SRY-box containing gene 2), and *REX-1* (also referred to as *ZFP42*, zinc finger protein 42) in srFGSCs using RT-PCR analysis. The results showed that the cells express *DDX4*, *IFITM3*, *OCT4*, *STELLA*, *DAZL*, *BLIMP-1*, and *REX-1* (see below). Immunofluorescence analysis confirmed the expression of DDX4, OCT4, IFITM3, and BLIMP-1 (Fig. 1G). Collectively, these results suggested that the srFGSC line was consistent with the previously reported morphology and germline lineage properties of human FGSCs obtained from healthy reproductive-age women^13^.

### Isolation, culture, and characterization of human FGSCs from follicular aspirates

**Figure 2:**
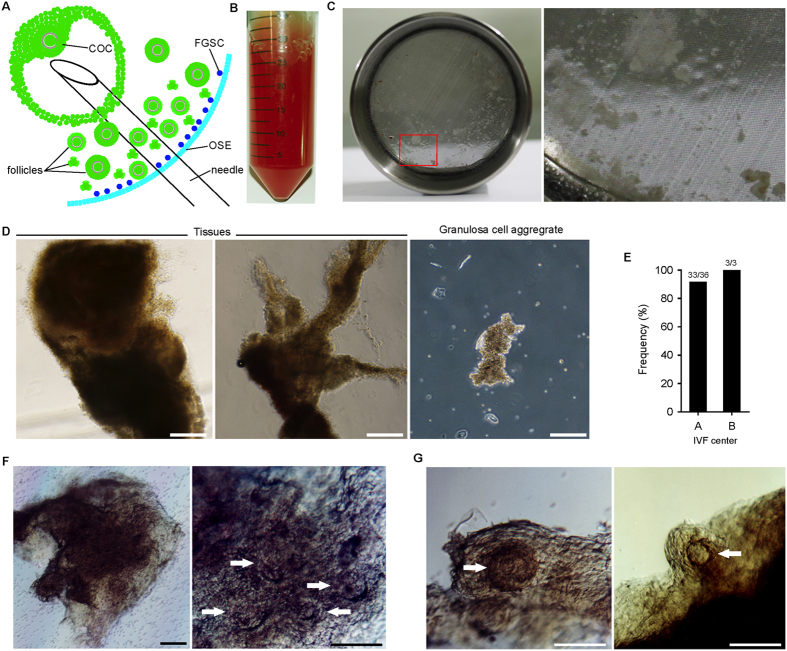
Scarce ovarian tissues exist in follicular aspirates. (**A**) Scheme of transvaginal oocyte retrieval performed in IVF centers showing preantral follicles and FGSCs possibly captured along with the mature oocytes. COC, cumulus-oocyte complex; OSE, ovarian surface epithelium. (**B**) Representative image of FAs in a 50-mL tube obtained from IVF centers. (**C**) A 30-μm nylon cell strainer with ovarian tissues and granulose cell aggregates from FAs (left). The red box is enlarged on the right. (**D**) Representative images of ovarian tissues (left, middle) and granulosa cell aggregates (right) captured from FAs showing distinct morphologies. (**E**) Proportion of samples containing ovarian tissues. (**F**) Bright-field images of human ovarian cortical tissue containing preantral follicles captured from FAs. The left image is enlarged on the right showing preantral follicles (white arrows). (**G**) After 2 days of culture *in vitro*, the preantral follicles (white arrows) were visible. Scale bars: 50 μm (**D**), 100 μm (**F**,**G**).

In an attempt to achieve faFGSCs, FAs after oocyte retrieval were obtained from 2 independent IVF centers. We found small pieces of ovarian tissues from FAs in both IVF centers after removing blood and single somatic cells with a 30-μm nylon cell strainer (Fig. 2A–D). These tissues were observed (91.7% and 100% of samples, respectively) in both IVF centers (Fig. 2E). Furthermore, some of these ovarian tissues included several small preantral follicles (Fig. 2F). When we cultured these follicles for 2 days, they grew and their follicular structures were more clearly seen (Fig. 2G). Based on the information^30^ and results above, we reasoned that the ovarian tissues (including ovarian cortexes) containing FGSCs were possibly captured through the ovary puncture and collected in FAs along with the mature oocytes (Fig. 2A).

**Figure 3:**
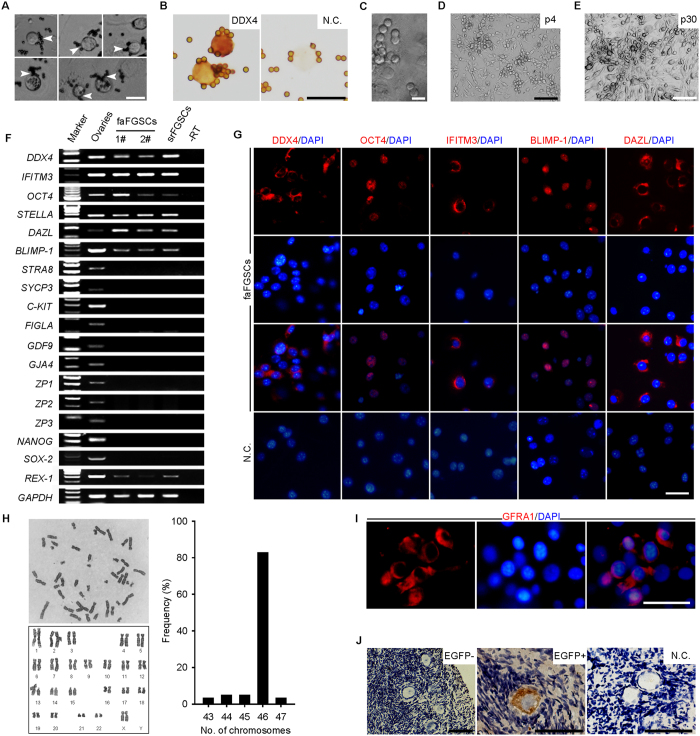
Isolation and characterization of human faFGSCs from follicular aspirates. (**A**) Representative images of DDX4^+^ cells from FAs after 2 weeks of culture. The white arrowheads indicate the magnetic beads. (**B**) The DDX4^+^ cells were confirmed by immunocytochemical analysis with the antibody against C-terminal of DDX4. (**C**) A representative morphology of mitotic faFGSCs. (**D**) A representative morphology of faFGSCs at passage 4 (p4). (**E**) A representative morphology of faFGSCs at passage 30 (p30). (**F**) RT-PCR analysis human ovarian tissues (28 years old), faFGSCs (cell line 1# and 2#), and srFGSCs. Lane 1, 100-bp DNA markers. –RT, PCR of RNA sample without reverse transcription. *GAPDH* is sample loading control. (**G**) The faFGSC line was detected by immunofluorescence analysis with the antibodies against DDX4, OCT4, IFITM3, BLIMP-1, and DAZL. (**H**) Karyotype analysis of the faFGSC line by G banding showing normal 46, XX and the distribution of metaphase spreads with different chromosome numbers (right). n = 59. (**I**) The faFGSC line was detected by immunofluorescence analysis with the antibody against GFRA1. (**J**) Immunohistochemistry staining of EGFP-positive cells (EGFP^+^, brown) surrounded by smaller EGFP^−^ cells in adult human ovarian cortical tissues injected by EGFP^+^ faFGSCs and xenografted into nude female mice for 2 weeks. The nuclei were counterstained with hematoxylin (blue). Full-length gels in F are presented in Supplementary Fig. 5. N.C. in (**B**,**G**,**J)** are omission of the primary antibody. Scale bars: 20 μm (**A**–**C**), 50 μm (**G**, **I**,**J**), 100 μm (**E**), 200 μm (**D**).

We subsequently isolated FGSCs from these ovarian tissues using two-step enzymatic digestion. Because of the small amount of faFGSCs in the samples, we cultured the isolated cells on a feeder layer for 2 weeks followed by purifying with MACS. In keeping with the results from srFGSCs, we observed that round or ovoid cells were bound to magnetic beads and displayed a morphology and size similar to srFGSCs (Fig. 3A). These purified cells were characterized by determining the expression of DDX4 using immunocytochemistry (Fig. 3B). The DDX4^+^ cells were then cultured further on a feeder layer. During the first week of culture after purification, the cells formed spherical or grape-like clusters consisting of 4–8 cells (Fig. 3C). As culturing continued, the clusters increased in number (Fig. 3D). After 10 weeks in culture, proliferation of the cells became rapid and stable and required passaging at confluence every 5–7 days with a split ratio of 1:3. We also found that faFGSCs, like srFGSCs, could be thawed after cryopreservation and re-established in cell culture with high efficiency (Fig. 3E). At the time of writing, the faFGSCs had been passaged more than 60 times.

To characterize these faFGSC lines, we applied RT-PCR and immunofluorescence analysis to determine their gene expression profile. RT-PCR analysis revealed that the expression of *DDX4*, *IFITM3*, *OCT4*, *STELLA*, *DAZL*, *BLIMP-1*, and *REX-1* was detectable in faFGSCs (line 1# and 2#) and srFGSCs. By contrast, meiosis (*STRA8* and *SYCP3*)-, oocyte (*C-KIT*, *FIGLA*, *GDF9*, *GJA4*, *ZP1*, *ZP2* and *ZP3*)-, and pluripotency (*NANOG* and *SOX-2*)-related genes were not detectable (Fig. 3F). Immunofluorescence analysis validated the expression of DDX4, OCT4, IFITM3, BLIMP-1, and DAZL in faFGSCs (Fig. 3G). Dual immunofluorescence analysis of bromodeoxyuridine (BrdU) incorporation and DDX4 expression demonstrated their mitotic activity (Supplementary Fig. S1A). Karyotyping analysis of faFGSCs lines by G-Banding showed a normal karyotype of 46, XX in 83% of metaphase spreads (Fig. 3H). In addition, faFGSCs were positive for alkaline phosphatase staining (n = 4 experiments), although their intensity seemed weaker than that of human embryonic stem cells (ESCs) (Supplementary Fig. S1B), which is consistent with our previous observations^11^,^14^,^19^,^24^. We also observed the expression of GDNF (glial cell-derived neurotrophic factor) receptor GFRA1 (GDNF family receptor alpha-1), one key marker involved in SSCs self-renewal^2^, in faFGSCs (Fig. 3I). We next examined whether faFGSCs could differentiate into oocytes in human ovarian tissues, considered to be the most stringent test for function. We injected enhanced green fluorescent protein positive (EGFP^+^) faFGSCs which labelled using lentivirus (see Supplementary Methods) into ovarian cortical tissues removed from 26- to 31-year-old patients with an ovarian cyst (n = 3, see Supplementary Methods), and subsequently subcutaneous xenografted tissues into adult nude female mice. Recipient ovarian tissues were collected 2 weeks after transplantation. Consistent with a previous report^13^, EGFP^+^ cells surrounded by one layer of EGFP^−^ somatic cells, resembling chimeric follicles, could be identified by immunohistochemistry (Fig. 3J). Collectively, these results demonstrated that scarce ovarian tissues in FAs could serve as an alternative source of FGSCs that share similar properties with srFGSCs.

### Spontaneous differentiation of candidate human faFGSCs *in vitro*

**Figure 4:**
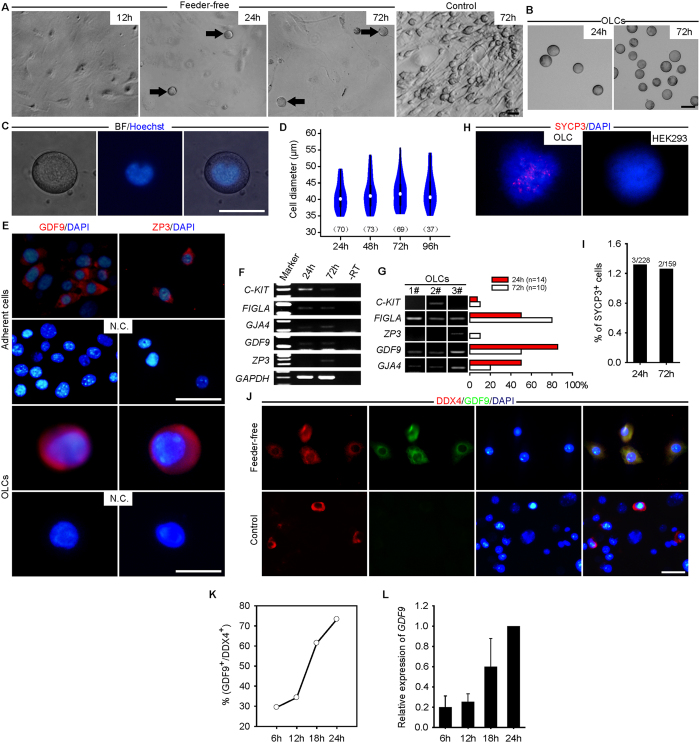
Spontaneous differentiation of candidate faFGSCs *in vitro*. (**A**) OLCs (black arrows) appeared when faFGSCs cultured under a feeder-free condition begin at 24 h but not at 12 h or cultured similarly but on a feeder layer (control). (**B**) Groups of OLCs floating in the medium collected at 24 h and 72 h after faFGSCs cultured under a feeder-free condition. (**C**) Representative bright-field (left), Hoechst 33342 (middle), and merged (right) images of one live OLC. (**D**) Violin plots showing the distribution of the diameter of OLCs formed by faFGSCs after seeding with 2 × 10^4^ cells in each culture well. Numbers in brackets denote the number of OLCs measured. (**E**) Adherent germ cells (upper panel) and OLCs (lower panel) were detected by immunofluorescence analysis with the antibodies against GDF9 and ZP3 at 72 h. (**F**) RT-PCR analysis of mRNAs encoding oocyte-related genes at 24 h and 72 h. (**G**) Single-cell RT-PCR analysis of mRNAs encoding oocyte-related genes in OLCs. Proportion of each gene is shown on the right. (**H**) Meiotic spread assays of SYCP3 expression in OLCs collected in medium at 72 h (left) and in HEK293 cells (right). (**I**) Percentage of cells showing punctate SYCP3 staining. (**J**) Representative image of dual immunofluorescence analysis for DDX4 and GDF9 at 18 h when faFGSCs cultured under different conditions. (**K**) Quantification of GDF9^+^ cells in DDX4^+^ cells at 6–24 h after faFGSCs cultured under a feeder-free condition. (**L**) Relative expression of mRNAs encoding *GDF9* in faFGSCs cultured under a feeder-free condition. Data are presented as mean ± SEM for three experiments. Full-length gels in F and G are presented in Supplementary Fig. 5. N.C. in (**E)** is omission of the primary antibody. Scale bars: 25 μm (**J**), 50 μm (**A**–**C**,**E**).

To determine the differentiation potential of faFGSC lines, we withdrew the feeder layer in cultures. After 12 hour (12 h) in culture at low density, faFGSCs showed extended pseudopodia. However, at approximately 24 h of culture, some of the faFGSCs gradually detached from the growth surface and large spherical cells emerged, whereas no obvious large cell was observed when faFGSCs were cultured on the feeder layer (Fig. 4A, Supplementary Fig. S2A and Supplementary Video S1). Time-lapse analysis revealed that adherent faFGSCs heterogeneously transformed into suspended large spherical cells (Supplementary Fig. S2B). These large spherical cells were morphologically identical to oocytes but lacked zona pellucida and diffuse chromatin (hereinafter referred to as oocyte-like cells, OLCs) (Fig. 4A–C). In line with previous report^13^, we found that the size of OLCs was similar (35–56 μm in diameter) during spontaneous differentiation (Fig. 4D). To characterize the OLCs, RT-PCR and immunofluorescence analysis were used to determine the expression of five oocyte related genes, including *C-KIT*, *FIGLA*, *GJA4*, *GDF9*, and *ZP3* in OLCs. The results showed that all of them were expressed in OLCs at 72 h, although *ZP3* was not detectable at 24 h (Fig. 4E,F). Single-cell RT-PCR analysis revealed heterogeneous expression of oocyte-related genes in individual OLCs, indicating their different developmental stages and cellular identities (Fig. 4G). To determine the meiosis chromosome segregation pattern of OLCs, spreads generated from the suspended cells were stained with SYCP3. Approximately 1.3% and 1.2% of punctuate SYCP3, probably corresponding to that of the synaptonemal complexes at the preleptotene stage of meiotic prophase I, were detected at 24 h and 72 h, respectively. No elongated SYCP3 was identified in the OLCs. HEK293 cells examined did not show any staining with SYCP3 (Fig. 4H,I). The low proportion of SYCP3^+^ cells in suspended cells suggests that meiosis is highly inefficient during spontaneous differentiation. To further probe the process of spontaneous differentiation of faFGSCs *in vitro*, dual immunofluorescence analysis on DDX4 and GDF9 were performed at 6-h time intervals over 24 h after plating. We found that the proportion of GDF9^+^ cells gradually increased from 29% at 6 h to 73% at 24 h, as confirmed by quantitative RT-PCR (qRT-PCR) analysis, suggesting that more than one fourth of FGSCs started spontaneous differentiation after 6 h of culture without the feeder layer (Fig. 4J–L). Collectively, these findings demonstrated that faFGSCs have the potential to differentiate into oocytes.

### Retinoic acid and granulosa cells regulate the growth and development of oocytes from candidate human faFGSCs *in vitro*

**Figure 5:**
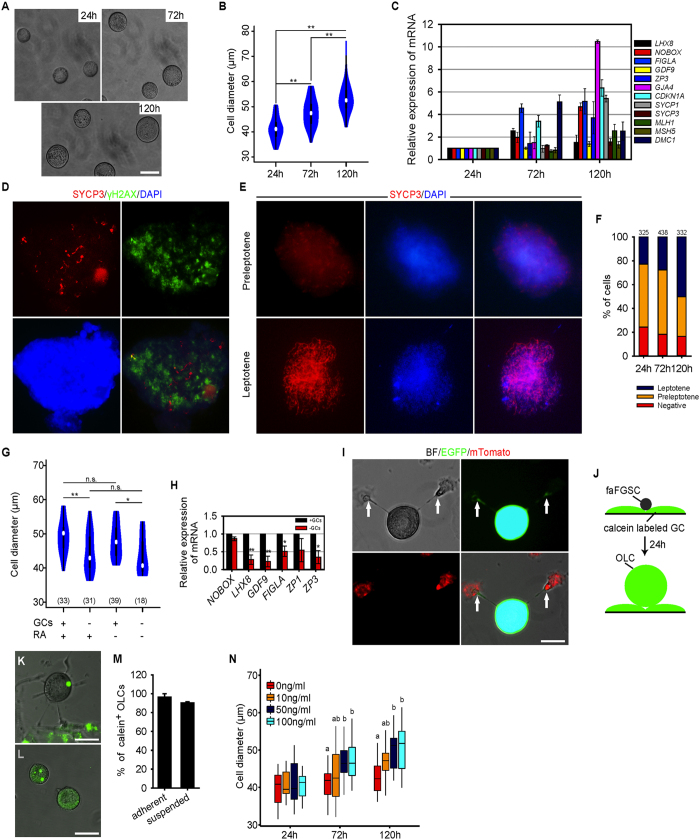
Retinoic acid and granulosa cells regulated the development of OLCs differentiated from candidate faFGSCs *in vitro*. (**A**) Groups of OLCs collected from medium when faFGSCs cultured under RA-supplemented and GC monolayers. (**B**) Violin plots showing the distribution of the diameter of OLCs differentiated by faFGSCs. (**C**) Relative expression of mRNAs encoding oocyte- and meiosis-related genes. (**D**) Meiotic spread from OLCs; dual immunofluorescence analysis of SYCP3 and γH2AX is shown. (**E**) Meiotic spread assays of SYCP3 expression in OLCs. (**F**) Percentage of cells showing preleptotene and leptotene SYCP3 staining. Numbers labeled denote the number of meiotic spreads counted for each sample. (**G**) Violin plots showing the distribution of the diameter of OLCs differentiated by faFGSCs at each condition. (**H**) Relative expression of mRNAs encoding oocyte-specific genes in faFGSCs cultured on GC monolayer (+GCs) or not (–GCs). (**I**) Representative morphologies of tentacle-like structures observed between OLCs (EGFP, green) and GCs (mTomato, red). White arrows indicate the connection sites. (**J**) Scheme of calcein transfer from GCs to OLCs. (**K**) Representative morphology of OLC growing on the plate labeled with calcein at 24 h. (**L**) Representative morphologies of OLCs in the medium labeled with calcein at 24 h. (**M**) Proportion of adherent and suspended OLCs labeled with calcein. (**N**) Boxplots showing the distribution of the diameter of OLCs differentiated by faFGSCs cultured under 0 ng/mL, 10 ng/mL, 50 ng/mL, and 100 ng/mL SCF at 24 h, 72 h, and 120 h. n = 294 in total. Numbers in brackets in (**G**) denote number of OLCs measured. Data in (**C**,**H**) are mean ± SEM for three experiments. Data in (**B**,**G**,**N)** were analyzed using one-way analysis of variance followed by the Tukey’s *post hoc* test. Data in (**H**) were analyzed using a Student’s t-test. *Indicates P < 0.05, **indicates P < 0.01. No significant difference (n.s.) indicates P > 0.05. Different characters in (**N)** represent statistically significant differences (P < 0.05). Scale bars: 50 μm (**A**,**I**,**K**,**L**).

The inefficient growth and development of oocytes observed in feeder-free conditions led us to improve the differentiation condition by adding RA and GCs to cultures. In accordance with the above observations, OLCs were released from the growth surface at 24 h (Fig. 5A, Supplementary Fig. S3A,B). However, no OLCs were observed in GC cultures (Supplementary Fig. S3A). Surprisingly, the size of the OLCs was increased from 41 ± 1.8 μm (mean ± SEM) at 24 h to 52 ± 1.2 μm at 120 h (Fig. 5B). qRT-PCR analysis revealed that the transcripts of *LHX8* (LIM homeobox 8), *NOBOX* (NOBOX oogenesis homeobox), *FIGLA*, *GDF9*, *ZP3*, *GJA4*, *CDKN1A* (cyclin-dependent kinase inhibitor 1A, a mitotic inhibitor), *SYCP1*, *SYCP3*, *MLH1* (mutL homolog 1), *MSH5* (mutS homolog 5), and *DMC1* (DNA meiotic recombinase 1) were increased by 120 h (Fig. 5C). Dual immunofluorescence analysis detected the expression of SYCP3 and γH2AX in OLCs, suggesting that some of them have entered meiosis (Fig. 5D). Furthermore, meiotic spread assays detected elongated SYCP3, probably at the leptotene stage, in suspended OLCs (Fig. 5E). After prolonged RA induction, the proportion of elongated SYCP3 increased from 23% at 24 h to 50% at 120 h (Fig. 5F). To explore the mechanisms of faFGSC differentiation, faFGSCs were cultured under different conditions. The results showed that the groups with GCs were much larger than groups without GCs in OLC size. However, there was no difference in size of OLCs between groups with RA and without RA (Fig. 5G). Moreover, qRT-PCR analysis revealed that GCs enhanced the expression of oocyte specific genes including *NOBOX*, *LHX8*, *GDF9*, *FIGLA*, *ZP1*, and *ZP3* compared with the control in the absence of GCs (Fig. 5H). These results suggested that the growth of OLCs was owing to the supporting activity of the monolayer of GCs, or the GCs play an important role in growth of OLCs. To address this mechanism, we found that some OLCs made contact with GCs through tentacle-like structures (Supplementary Fig. S3C and Supplementary Video S2). Phalloidin staining for actin filaments showed that they were localized in the tentacle-like structures (Supplementary Fig. S3D). To verify, EGFP^+^ faFGSCs were cultured on a monolayer of GCs isolated from ROSA^mT-mG^ female mice that ubiquitously express membranous Tomato (mTomato). As expected, some EGFP^+^ OLCs growing on the plate bottom made contact with mTomato^+^ GCs through EGFP^+^ tentacle-like structures (Fig. 5I).

Gap-junctional intercellular communication (GJIC) is crucial in terms of oocyte growth and maturation^34^. GJA4 is the major connexin that builds the gap junctions between the oocyte and GCs^35^. Based on these reports, we detected expression of GJA4 in OLCs using immunofluorescence analysis. The result showed that the OLCs expressed GJA4 (Supplementary Fig. S3E). To further determine the function of GJIC between OLCs and GCs, we seeded faFGSCs onto GC monolayers pre-loaded with calcein-AM (a gap-junction-permeable fluorescent dye) (Fig. 5J). As expected, 24 h later, fluorescent dye was consistently detected in adherent (with tentacle-like structures) and suspended OLCs with high frequency (97% and 91%, respectively) (Fig. 5K–M). These results suggested that most OLCs have built functional gap junctions with surrounding GCs prior to their release from the growth surface. Apart from GJIC, paracrine factors secreted by GCs also promote the growth and development of oocytes. For example, SCF promotes gap junction-independent oocyte growth^36^. We therefore replaced GCs with exogenous SCF at different concentrations in the culture medium. As expected, SCF induced the majority of OLCs growth in a dose- and time-dependent manner (Fig. 5N). Thus, GCs facilitated the growth and development of OLCs by gap junctions and paracrine factors *in vitro*.

**Figure 6:**
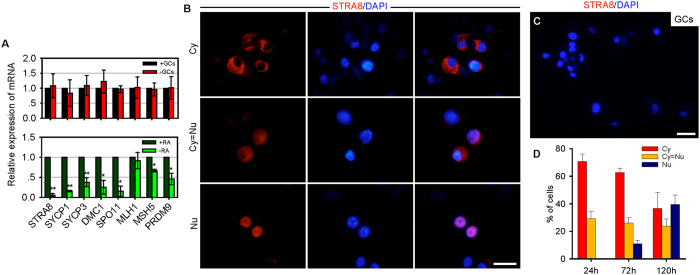
Retinoic acid induces the meiotic initiation of faFGSCs. (**A**) Relative expression of mRNA encoding meiosis-related genes in faFGSCs cultured onto GC monolayer (+GCs) or not (−GCs) or under RA-supplemented (+RA) or RA-free (−RA) conditions at 120 h. (**B**) Immunofluorescence analysis of STRA8 (left panel) in faFGSCs after RA treatment, showing heterogeneous distribution in the cytoplasm and nucleus. Cy, cytoplasm; Cy = Nu, cytoplasm and nucleus; Nu, nucleus. (**C**) GCs cultured under the RA-supplemented condition for 120 h staining STRA8 served as the negative control. (**D**) Quantification of the different patterns of STRA8 sub-cellular localization in faFGSCs after RA treatment at 24 h, 72 h, and 120 h. Data in (**A**,**D)** are mean ± SEM for three experiments. Data in (**A)** were analyzed using a Student’s t-test. *Indicates P < 0.05, **indicates P < 0.01. Scale bars: 50 μm (**B**,**C**).

To study the role of RA in faFGSC differentiation, we first determined the expression of transcripts of RA receptors, retinoid X receptors, and cellular RA-binding proteins in faFGSCs by RT-PCR analysis. The results showed that faFGSCs express *RARA*, *RXRA*, *RXRB*, and *CRABP1* (Supplementary Fig. S3F), suggesting that meiotic initiation in faFGSCs could be regulated by RA signaling via forming active RAR-RXR heterodimers^37^. Moreover, qRT-PCR analysis revealed that *STRA8*, *SYCP1*, *SYCP3*, *DMC1*, *SPO11*, *MSH5*, and *PRDM9* (PR domain-containing 9, a major determinant of meiotic recombination hotspots) were increased in faFGSCs after RA treatment compared with the control in the absence of RA (ethanol), but not improved by GCs (Fig. 6A). Immunofluorescence analysis of STRA8 in faFGSCs after RA treatment revealed a distinct intracellular localization. Furthermore, after prolonged RA induction, the proportion of STRA8 nuclear localization increased from 0% at 24 h to 39.4% at 120 h, whereas the proportion of cytoplasmic localization decreased from 70.8% at 24 h to 36.7% at 120 h (n = 3 experiments) (Fig. 6B–D). The translocation of STRA8 from the cytoplasm to the nucleus is perhaps important for meiotic initiation. Collectively, these data indicated that RA and GCs could initiate the meiotic process and regulate the growth and development of oocytes, respectively, from faFGSCs.

### GV oocytes generated by candidate human faFGSCs *in vitro*

**Figure 7:**
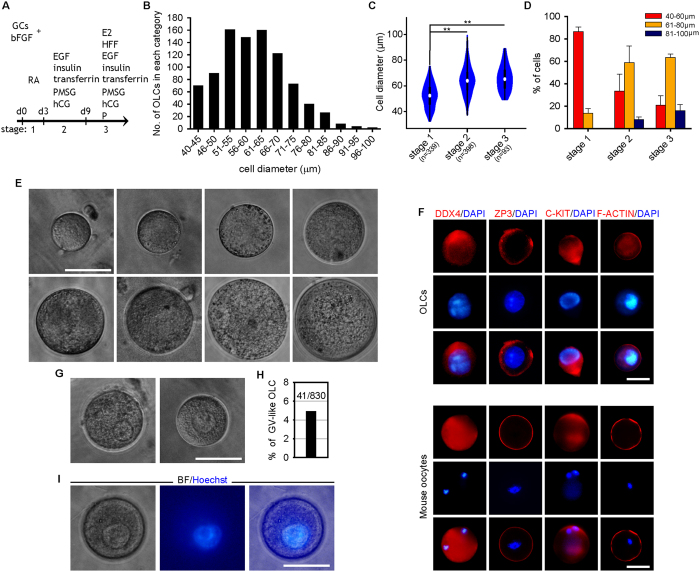
GV OLCs *in vitro* differentiated from candidate human faFGSCs. (**A**) Scheme showing the time course and culture conditions for the differentiation of faFGSCs into oocytes *in vitro*. (**B**) Distribution of OLCs of different diameters. n = 904 in total. (**C**) Violin plots showing the distribution of the diameter of OLCs formed by faFGSCs at different stages. Numbers in brackets denote the number of OLCs measured. (**D**) Proportion of 40–60 μm, 61–80 μm, and 81–100 μm diameter OLCs. Data are presented as mean ± SEM for three experiments. (**E**) Representative morphologies of OLCs observed during differentiation. (**F**) Immunofluorescence analysis of OLCs and mouse oocytes for DDX4, ZP3, C-KIT, and F-ACTIN. (**G**) Representative morphologies of GV-like OLCs. (**H**) Proportion of GV-like OLCs observed during differentiation. (**I**) Representative bright-field (left), Hoechst 33342 (middle), and merged (right) images of GV-like OLC. Mouse oocytes in (**F)** are controls. Data in (**C)** were analyzed using one-way analysis of variance followed by the Tukey’s *post hoc* test. **indicates P < 0.01. Scale bars: 50 μm (**E**–**G**,**I**).

To further explore human oogenesis *in vitro*, we developed a three-stage protocol to differentiate oocytes from faFGSCs. First, faFGSCs were cultured onto a GC monolayer for 3 days in bFGF and RA medium, and thereafter, for 6 days in the presence of bFGF, EGF, insulin, transferrin, PMSG (pregnant mare’s serum gonadotropin), and hCG (human chorionic gonadotropin). Subsequently, E2 (17-β-estradiol), P (progestogen), and HFF (human follicular fluid) were added to the medium for 3–6 days of culture (Fig. 7A). We observed that OLCs consistently emerged from the growth surface during the differentiation progress. Measurements of diameter revealed a large number of OLCs ranged from 51 μm to 70 μm in diameter, whereas only a small proportion of them were over 80 μm with the largest reaching 99 μm (Fig. 7B). The mean diameter of OLCs (n = 830) increased from 52.5 ± 0.5 μm (mean ± SEM) at stage 1 to 65.8 ± 1 μm at stage 3 (Fig. 7C–E). Immunofluorescence analysis on single OLCs revealed the expression of DDX4, ZP3, and C-KIT. Actin filaments stained with phalloidin showed that they were localized beneath the cell membrane and in the cytoplasm around the nucleus of OLCs. As positive controls, the oocytes from mouse ovaries were used (Fig. 7F). We also occasionally found some OLCs (4.9%) had a GV-like structure (Fig. 7G,H). Hoechst 33342 staining of live OLCs demonstrated that they contained one nucleus with diffuse chromatin (Fig. 7I)

To exclude the possibility that the observed OLCs differentiated from mouse FGSCs that were already present in the GCs preparation, we repeated the experiment using EGFP^+^ faFGSCs that were sorted by MACS using the antibody against the C-terminal of DDX4. Consistent with the prior results, we detected EGFP^+^ OLCs at various stages and some of them were still associated with the magnetic beads (Supplementary Fig. S4). Collectively, these results demonstrated that human GV oocytes could be developed from faFGSCs *in vitro* under defined culture conditions.

## Discussion

In general, human ovarian cortical tissues are obtained during caesarean section or laparoscopic surgery for infertility investigations, or in patients undergoing oophorectomy for various gynecological disorders. FAs are routinely produced in IVF centers worldwide and often discarded after oocyte retrieval. They contain numerous types of somatic cells including GCs, theca cells, blood cells, and some vaginal, ovarian surface epithelial cells, as well as oocytes. Whether FGSCs could be achieved from this by-product of ART attracts our attention and interest. In this study, we found scarce ovarian tissues in FAs and developed a simple and universal method of separating these tissues from FAs. Furthermore, we have isolated, purified, and achieved long-term culture of human FGSCs from these tissues. The results show for the first time that these ovarian tissues could provide an alternative source of human FGSCs. Considering the successful establishment of FGSC lines from scarce ovarian tissues in FAs reported here and the fact that stimulation protocols involve in each ART cycle prior transvaginal oocyte retrieval, it is necessary to evaluate whether the stimulation protocols have effects on FGSCs in further study.

We used MACS to purify faFGSCs after *in vitro* proliferation owing to the scarcity of samples in FAs. To confirm whether our cultures are naïve FGSCs in FAs, we compared their expression profiles, growth characteristics, and differentiation potentials with srFGSCs expanded from the ovarian cortex surgically removed from patients, and demonstrated that they share similar properties. Additionally, we also confirmed that the sorted cells using MACS coupled the antibody against to the C-terminal of DDX4 were germline cells (Figs 1D,E, 3B and Supplementary Fig. S4A), which consistent with the previous observations from our peers and us. These observations imply that our separation protocols and culture methods are suitable for establishing FGSC lines even from scarce ovarian cortical tissues.

Very recently, Hernandez *et al*. claimed that the purified cells using FACS coupled with the antibody against the C-terminal of DDX4 were non-germline cells despite they support the existence of FGSCs^38^. Zhang *et al*. also claimed that DDX4-specific antibody in the FACS did not select for DDX4-expressing cells and the sorted cells were not functional GSCs^39^. In response to their claims, Woods *et al*. noticed that the large range in relative frequency of antibody-positive (Ab + ve) cells obtained from human ovaries (4.5–24% positive) and rhesus macaque ovaries (2.5–50.6%) reported by Hernandez *et al*. may be caused by improper operation of FACS. They also proved this method is feasible by providing new data that FGSCs could be purified from ovaries of female baboon (*Papio anubis*) during peak reproductive life using FACS coupled with this antibody^40^. In their reports, Hernandez *et al*. failed to detect the expression of DDX4 in dispersed unsorted ovarian cell fractions that should contain oocytes expressing DDX4^38^. Similarly, Hernandez *et al*. and Zhang *et al*. also failed to detect the expression of DDX4 in antibody-negative (Ab−ve, also named DDX4-negative by Zhang *et al*.) cell fractions^38^,^39^. Due to zona pellucida (a thick extracellular coat that surrounds the oocytes) can build a barrier between DDX4 antibody and cell, oocytes with zona pellucida would present in Ab−ve fractions despite the large oocytes were removed using a 70 μm cell strainer, which demonstrated by the results from White *et al*. that *Ddx4* along with oocyte specific markers (*Nobox*, *Zp3* and *Gdf9*) could be detected in Ab-ve fractions^13^. These discrepancies indicate that the dispersed unsorted ovarian cells generated by Hernandez *et al*. and Zhang *et al*. are quite different from those generated by White *et al*. despite the same tissue-dissociation methods were used. The tissue architecture of mammalian ovaries is varied with species and ages. For example, dense ovarian stroma exists in older ovaries^41^. It is maybe not suitable to disperse ovarian tissues from older patients (aged between 30–60 years and average age 32.1 ± 3.7 years used in Hernandez *et al*. and Zhang *et al*., respectively) using a GentleMACS tissue dissociator according to the programs which suitable to disperse ovarian tissues from younger patients [between 22-33 (28.5 ± 4.0) years of age]^42^. The ovaries with dense connective-tissue matrix may result in the spurious redistribution of epitopes recognized by DDX4-specific antibodies^43^. It is not appropriate to draw a conclusion that the antibody against the C-terminal of DDX4 is not suitable to sort FGSCs according to the result that no germline cell was sorted from a group of cells not containing germline cells.

In this study, we found that oocytes with a diameter of 35–56 μm differentiated from faFGSCs and this was a cell-autonomous process without an STO feeder layer, which is consistent with a previous report demonstrating that mouse and human FGSCs could spontaneous differentiate into oocytes in mouse embryonic fibroblasts -free conditions^13^. These results indicate that the feeder cells play a crucial role in maintaining the undifferentiated state of FGSCs, and the FGSCs own identity of germline lineage. The differentiation potential of faFGSCs was assessed by various approaches, including morphological observation, single cell analysis, RT-PCR, immunofluorescence analysis, and meiotic spread assay. Genes for oocyte markers were expressed in the germ cells differentiated from faFGSCs. Heterogeneous expression of oocyte-related genes in individual OLCs detected by single-cell RT-PCR may account for the heterogeneous transformation from adherent faFGSCs. Observations of OLCs with highly inefficient meiosis, similar to the study on *Stra8*-deficient mice where oocyte-like structures are formed but meiosis was not triggered, revealed the genetic dissociation between female germ cell differentiation and meiosis^44^. Unlike oocytes differentiated by pluripotent stem cells including ESCs and induced pluripotent stem cells, no follicle-like structure was observed during faFGSC differentiation *in vitro* despite extensive searching, which is consistent with previous results^13^,^24^. This is a shared characteristic with the germline lineage commitment and differentiation of SSCs.

In this study, we co-cultured faFGSCs onto a monolayer of GCs from juvenile mice to mimic the follicular microenvironment that results in oocyte growth and development. Coordinated communication between oocytes and their surrounding somatic cells is essential for follicular development. Studies on mice demonstrated that oocyte growth could be achieved on monolayers of preantral GCs, but not on other communication-competent somatic cells^45^,^46^,^47^. Similarly, in this study, we also found that co-cultured GCs result in the growth and development of OLCs demonstrated by the increased cell diameter and mRNA transcripts of oocyte-related genes. The formation of tentacle-like structures between some OLCs and surrounding GCs was another interesting phenomenon that we observed when faFGSCs were cultured onto GC monolayers. Very little is known about the molecular mechanisms underlying the formation of primordial follicles, which occurs in the human ovary during fetal life. The tentacle-like structures may help us to understand the mechanisms of primordial follicle formation. GJIC is important for oocyte growth and development. Functional GJIC was determined by examining the transfer of calcein, a gap-junction-permeable dye, from GCs to OLCs with high efficiency. Thus, gap-junctional exchange of small molecules such as ATP, sodium, chloride, sugars, amino acids, calcium ions, and cAMP between two compartments may facilitate the development of OLCs. SCF, which is secreted by GCs, can promote gap junction-independent oocyte growth^36^. We also found that exogenous SCF in the culture medium could induce the majority of OLC growth in a dose- and time-dependent manner. Thus, GCs promote OLC growth and development by means of GJIC and paracrine secretion factors.

Mitosis/meiosis transition marks an important event for germ cell development. STRA8, induced by RA, is one master gene controlling meiosis induction in human ovaries around 10 wpf (weeks post fertilization). Our results demonstrate that RA can trigger the meiotic process of faFGSCs by examining the meiotic-related genes and the synaptonemal complexes. STRA8 function is required for germ cells to transition from preleptotene to leptotene. The expression of *DMC1* and *SPO11* suggests that OLCs undertake meiotic recombination. These results suggest that the meiotic process was promoted onwards by their own regulatory networks. It was reported previously that STRA8 could shuttle between the cytoplasm and nucleus via nuclear localization and export signals^48^. Here, we also found this distinct intracellular localization and the dynamic changes of these two compartments with prolonged RA induction, but the functions of cytoplasmic and nuclear STRA8 were unknown. Previous studies demonstrated that STRA8 directly binds to DNA and possesses transcriptional activity^48^,^49^. By using a yeast two-hybrid system, Zheng *et al*. screened six STRA8 candidate interaction proteins and thus concluded that chromatin assembly/modification and transcriptional regulation might be involved in its functions^50^. It is postulated that STRA8 may have different functions in the cytoplasm and nucleus during the initiation of meiosis.

The protocol modified from our previous report^24^ enabled us to mimic human oogenesis and achieve GV oocytes from faFGSCs *in vitro*, although the frequency (about 5%) needs to be improved. To our knowledge, this is the first observation of human GV oocytes developed from FGSCs. Differentiating FGSCs into GV oocytes *in vitro* is an important step for exploring the mechanisms of human prenatal oogenesis and clinical applications. In view of developmental differences between rodent and human in oogenesis and the fact that 10–40 wpf human embryos are excluded from investigations, our differentiation scheme may represent a model for tracking human oogenesis and exploring the root causes of female factor infertility. To date, complete oogenesis *in vitro* that provides human viable oocytes has not yet been accomplished. Thus, further work based on this system is needed to ask whether these developed OLCs behave real oocytes that can be fertilized.

Our findings may have practical implications for both clinical applications and basic science. For instance, the ability to achieve FGSCs from scarce ovarian tissues that exist in FAs would provide a new strategy of fertility preservation for women of reproductive age with cancer. Before chemo- or radio-therapy, FGSCs isolated from a small amount of ovarian cortical tissues collected using an ultrasound-guided transvaginal needle, could be cultured and cryopreserved *in vitro*. After treatment, FGSCs would be transplanted back into the patient’s ovaries for the production of oocytes. Additionally, after development of the necessary techniques, an unlimited numbers of functional oocytes from faFGSCs could be applied in future ART such as cytoplasmic transfer. Our *in vitro* differentiation scheme represents a novel strategy for exploring early stages of human oogenesis and primordial follicle formation, which is not possible because 10–40 wpf human embryos are excluded from investigations, as well as the root causes of female factor infertility.

In summary, we developed a strategy for establishing human FGSC lines from scarce ovarian cortical tissues (incorporated tissues in the puncture needle) exist in the “waste” FAs. The cell lines have characteristics of GSCs involved in the gene expression profile, growth characteristics, and a normal karyotype consistent with that of srFGSCs. Furthermore, the FGSCs have developmental potentials including spontaneous differentiation into oocytes, communicating with GCs, entering meiosis, as well as forming chimeric follicles in human ovarian cortical tissues xenografted into adult immunodeficient female mice. Finally, we also developed a strategy for differentiating FGSCs into GV oocytes *in vitro* and revealed their developmental mechanisms. The mechanisms including (i) GCs facilitated the growth and development of germ cells from faFGSCs by gap junctions and paracrine factors *in vitro*; (ii) RA could initiate the meiotic process of germ cells from faFGSCs via initiating STRA8 protein expression *in vitro*. Collectively, several lines of evidence presented here demonstrated that human FGSCs lines could be established from scarce ovarian tissues in FAs. Our study not only provides a new approach to obtain human FGSCs for medical treatment, but also opens several avenues to investigate human oogenesis *in vitro*.

## Methods

### Mice

Three-week-old CD1 wild-type and *B6.129(Cg)-Gt(ROSA)26Sor*^*tm4(ACTB-tdTomato,-EGFP)Luo*^*/J* (referred to as ROSA^mT-mG^) female mice, and 6-week-old nude female mice were used in this study. All mouse experiments were approved by the Institutional Animal Care and Use Committee (IACUC) at Shanghai Jiao Tong University, Shanghai, China [SYXK (Shanghai 2007-0025)] and all experiments were carried out in accordance with the approved protocols.

### Human Samples

With written informed consent, ovarian cortical tissues were surgically removed from seven adult patients aged between 26 and 43 (30 ± 2.26, mean ± SEM) years. Five patients were undergoing partial oophorectomy for an ovarian cyst; one patient was undergoing a hysterectomy and oophorectomy for cervical carcinoma not involving the ovaries; the other patient was undergoing partial oophorectomy for an ectopic pregnancy. The clinical data and applications are summarized in Table 1. The ovarian cortical layer was carefully removed for cryopreservation and/or cell isolation as previously described^11^,^51^.

With written informed consent, FAs were obtained from patients aged between 30 and 35 years. After oocyte retrieval, the remaining FAs from two to six different patients were collected into sterilized centrifuged tubes and transported to the laboratory within 2 h at room temperature for cell isolation.

The human study was approved by the Investigation Review Committee of Obstetrics and Gynecology Hospital, Fudan University, Shanghai; The First People’s Hospital of Chenzhou, Hunan Province, and Shanghai First Maternity and Infant Hospital affiliated to Tong Ji University and all patients provided written informed consent. All experiments were carried out in accordance with the approved protocols.

### Isolation of srFGSCs and faFGSCs

For isolation of srFGSCs, ovarian cortical tissues (approximately 0.1 g) were dissociated by mincing followed by a two-step enzymatic digestion method that has been described previously^11^,^24^. Briefly, the dissected ovarian tissues were placed in Hank’s balanced salt solution (HBSS) without calcium or magnesium containing collagenase IV (1 mg/mL; Sigma), followed by incubation at 37 °C with gentle agitation for approximately 20 min. After centrifugation at 300 g for 5 min and washing in HBSS, the ovarian tissues were placed in HBSS containing 0.5 mM EDTA and 0.05% trypsin at 37 °C with gentle agitation for 2–5 min. When most of the cells were dispersed, trypsin was neutralized by adding 10% fetal bovine serum (FBS, Hyclone). The suspension was centrifuged at 300 g for 5 min and the supernatant was carefully removed from the pellet. The pellet was resuspended and clumps of cells were removed by passing the suspension through a 70-μm nylon cell strainer. For MACS, sheep anti-rabbit IgG conjugated magnetic beads (Dynabeads, 112.03D) and goat anti-rabbit IgG conjugated microbeads (Miltenyi Biotec, 130-048-602) were pre-coated with a rabbit polyclonal DDX4 antibody (Abcam; ab13840) according to the methods described previously^11^ and the manufacturer’s instructions, respectively. DDX4-positive cells were separated by magnetic separation, according to the manufacturer’s instructions.

For isolation of faFGSCs, pooled FAs from two to six patients were passed through a 30-μm nylon cell strainer and rinsed with HBSS several times until no blood cells were visible. Small pieces of ovarian tissues were collected and placed in HBSS containing 1 mg/mL collagenase IV, then incubated at 37 °C with gentle agitation for 5 min. The ovarian tissues were then washed in HBSS, placed in HBSS containing 0.5 mM EDTA and 0.05% trypsin, and incubated at 37 °C for 2–3 min. When most of the cells were dispersed, trypsin was neutralized by adding 10% FBS. The suspension was centrifuged at 300 g for 5 min and the supernatant was carefully removed from the pellet. The pellet was resuspended and cultured on inactivated an STO (SIM Thioguanine/Ouabain-resistant mouse fibroblast cell line, 5 × 10^4^ cells/cm_2_, ATCC) feeder layer for 2 weeks followed by MACS using a DDX4 antibody as mentioned above.

### Culture of srFGSCs and faFGSCs

The srFGSCs and faFGSCs culture system uses a mitotically inactivated STO feeder layer. STO cells were cultured in Dulbecco’s modified Eagle’s medium with high-glucose (Life Technologies), 1 mM non-essential amino acids (NEAA; Life Technologies), 10% FBS (Hyclone), and 6 mg/L penicillin (Sigma). The STO cells were treated with 10 μg/mL mitomycin C (Sigma) for 2–3 h, then washed several times in HBSS, digested to single cells, and plated on 0.1% (w/v) gelatin-coated wells of a 24-well plate. The culture medium for srFGSCs and faFGSCs consist of minimum essential medium α (MEMα), 10% FBS (Front), 1 mM sodium pyruvate (Sigma), 1 mM NEAA, 2 mM L-glutamine (Sigma), 0.1 mM β-mercaptoethanol (Sigma), 10 ng/mL human leukemia inhibitory factor (LIF; Santa Cruz Biotechnology, sc-4377), 10 ng/mL human epidermal growth factor (EGF; Peprotech, AF-100-15), 40 ng/mL human glial cell line-derived neurotrophic factor (GDNF; Peprotech, 450-10), 10 ng/mL human basic fibroblast growth factor (bFGF; Peprotech, AF-100-18B), and 6 mg/L penicillin. FGSCs were cultured on STO feeders in FGSC culture medium (500 μL per well). The medium was changed every 2–3 days and cells were subcultured using dispase II (Roche) every 5–8 days at a 1:1–1:3 dilution. All cultures were maintained at 37 °C in a 5% CO_2_-95% air atmosphere.

### BrdU incorporation

BrdU (50 μg/mL; Sigma) was added to faFGSC cultures for 5 h. The cultures were processed for immunofluorescence staining of BrdU as described below.

### Alkaline phosphatase and immunofluorescence staining

Alkaline phosphatase (AP) staining was performed using an Alkaline Phosphatase Detection kit (Sigma) according to the manufacturer’s instructions.

For staining of adherent cells, the cells in 48 plates were washed with 1X-concentrated phosphate-buffered saline (PBS), fixed in 4% paraformaldehyde (PFA) for 20 min at room temperature, washed twice with PBS, and incubated for 10 min at 37 °C in blocking buffer (PBS containing 10% normal goat (for DDX4, OCT4, IFITM3, BLIMP-1, DAZL, GDF9, GFRA1, STRA8, and BrdU) or horse (for ZP3) serum). Then, they were incubated overnight in a humidified chamber at 4 °C with one of the following: 1:500 dilution of a rabbit polyclonal anti-DDX4 antibody (Abcam; ab13840); 1:100 dilution of a mouse monoclonal anti-DDX4 antibody (Abcam; ab27591); 1:150 dilution of rabbit polyclonal anti-OCT4 (Santa Cruz Biotechnology; sc-9081); 1:200 dilution of a rabbit polyclonal anti-IFITM3 antibody (Abcam; ab15592); 1:100 dilution of a rabbit polyclonal anti-BLIMP-1 (ABGENT Crown; AP14521a); 1:100 dilution of rabbit polyclonal anti-DAZL (Abcam; ab128238); 1:100 dilution of rabbit polyclonal anti-GDF9 (ABclonal; A2739); 1:100 dilution of rabbit polyclonal anti-GFRA1 (ABclonal; A5373); 1:100 dilution of rabbit polyclonal anti-STRA8 (ORIGENE; TA321988); 1:150 dilution of a mouse monoclonal anti-BrdU antibody (Thermo scientific; clone BRD.3); 1:100 dilution of goat polyclonal anti-ZP3 (Santa Cruz Biotechnology; sc-23717). After washing twice with PBS, the cells were incubated at 37 °C for 30 min with a 1:150 dilution of tetramethylrhodamine isothiocyanate (TRITC) conjugated secondary antibody (goat anti-rabbit IgG or goat anti-mouse IgG or rabbit anti-goat IgG; ProteinTech) or a 1:150 dilution of fluorescein isothiocyanate (FITC) conjugated secondary antibody (goat anti-rabbit IgG or goat anti-mouse IgG; ProteinTech), then incubated at 37 °C for 20 min with 500 ng/mL 4’,6-diamidino-2-phenylindole (DAPI; Sigma). The cells were then mounted in anti-fade mounting medium. Images were obtained using a Leica DMI3000 B microscope and a Leica DFC550 digital camera, using fluorescein optics for TRITC, FITC, and ultraviolet optics for DAPI. Appropriate negative controls were performed with the primary antibodies omitted.

For staining of OLCs and mouse oocytes, the individual cells were collected from supernatants and fixed in 4% PFA for 20 min at room temperature. After fixation, the cells were incubated for 10 min at 37 °C in blocking buffer (PBS containing 10% normal goat (for GJA4, DDX4, GDF9, and C-KIT) or horse serum (for ZP3)). The cells were then incubated overnight in a humidified chamber at 4 °C with a 1:100 dilution of rabbit polyclonal anti-GJA4 (ABclonal; A2529); or a 1:500 dilution of a rabbit polyclonal anti-DDX4 antibody (Abcam; ab13840); or a 1:100 dilution of rabbit polyclonal anti-GDF9; or a 1:100 dilution of rabbit polyclonal anti-C-KIT (Santa Cruz Biotechnology; sc-5535); or a 1:100 dilution of goat polyclonal anti-ZP3. After washing with PBS, the cells were incubated at 37 °C for 30 min with a 1:150 dilution of TRITC-conjugated secondary antibody (goat anti-rabbit IgG or rabbit anti-goat IgG; ProteinTech), then incubated at 37 °C for 20 min with 500 ng/mL of DAPI and mounted as described above. Appropriate negative controls were performed with the primary antibodies omitted.

For staining of human ovarian cortex, ovarian tissues used for analysis were prepared as described previously^11^. Briefly, after dewaxing, antigen retrieval was performed using 0.05% trypsin in PBS for 20 min at 37 °C. The sections were then incubated for 15 min at 37 °C with blocking buffer (PBS containing 10% normal goat serum) and subsequently incubated overnight with a 1:200 dilution of rabbit polyclonal DDX4 and a 1:100 dilution of mouse monoclonal KI67 (Abcam; ab6526) in a humidified chamber at 4 °C. After washing in PBS, the sections were incubated at 37 °C for 30 min with a 1:150 dilution of TRITC conjugated secondary antibody (goat anti-rabbit IgG) and a 1:150 dilution of FITC conjugated secondary antibody (goat anti-mouse IgG), then incubated at 37 °C for 20 min with 500 ng/mL of DAPI and mounted as described above. Images were obtained with a Leica DM2500 microscope and a Leica DFC 550 digital camera.

### Lentivirus infection of faFGSCs

EGFP lentivirus was obtained from a commercially available service (GM100101-1, Genomeditech, Shanghai, China) and the lentivirus infection was performed according to the manufacturer’s instructions.

### Intraovarian EGPF^+^ faFGSCs injection and xenografting

Intraovarian EGFP^**+**^ faFGSCs injection and xenografting were performed as described previously^13^. Xenografts were removed 2 weeks after transplantation, fixed immediately in 4% PFA, paraffin wax embedded, and serially sectioned for immunohistochemistry using a mouse monoclonal antibody against EGFP. Briefly, after dewaxing, antigen retrieval was first performed using 0.05% trypsin in PBS for 20 min at 37 °C. Sections were then incubated for 10 min with 3% hydrogen peroxide in methanol to block endogenous peroxidase activity at room temperature, washed, and incubated in blocking buffer (PBS containing 10% normal goat serum) for 20 min at 37 °C. The sections were then incubated overnight with a 1:100 dilution of a mouse monoclonal anti-GFP antibody (CST; mAb #2955) in a humidified chamber at 4 °C. After washing with PBS, the sections were incubated at 37 °C for 30 min with a 1:150 dilution of HRP (horseradish peroxidase)-conjugated secondary antibody (goat anti-mouse IgG; Jackson ImmunoResearch), then rinsed and reacted with DAB (3,3’ Diaminobenzidine) regents. Sections were lightly counterstained with hematoxylin to visualize cell and tissue architecture. Images were obtained using a Leica DM2500 microscope and a Leica DFC 550 digital camera. Appropriate negative controls were performed with the primary antibody omitted.

### Isolation and culture of mouse granulosa cells

GCs were isolated and cultured as previously described with some modification^52^. Briefly, ovaries from 3-week-old CD1 and ROSA^mT-mG^ female mice were placed in HBSS, and GCs were released by manually puncturing ovaries with 25-gauge needles. After passing the suspension through a 30-μm nylon cell strainer, the GCs were cultured in Dulbecco’s modified Eagle’s medium with high-glucose containing 1 mM NEAA, 10% FBS, and 6 mg/L penicillin. The GCs between the second and fourth passage were used for faFGSCs differentiation.

### *In vitro* differentiation of faFGSCs

For studying the spontaneous differentiation of faFGSCs, the cells were trypsinized and 2 × 10^4^ of cells were plated on a 0.1% (w/v) gelatin-coated well of a 24-well plate. The cells were cultured in stem cell culture medium. The medium was changed daily.

For assessing the functions of RA and GCs during faFGSCs differentiation, the cells were trypsinized and 2 × 10^4^ cells were cultured on a GC monolayer per well of a 24-well plate. The cells were cultured in basic medium (MEM-α containing 15% FBS, 2 mM L-glutamine, 1 mM NEAA, 10 ng/mL human bFGF, 1 mM sodium pyruvate, 0.1 mM β-mercaptoethanol, and 6 mg/L penicillin) containing 2 μM RA (Sigma, R2625) dissolved in ethanol for 5 days. The control medium contained the vehicle (ethanol) alone. The medium was changed daily. For assessing the function of SCF during faFGSCs differentiation, 0 ng/mL, 10 ng/mL, 50 ng/mL, and 100 ng/mL SCF (Peprotech, 250-03) were added to the medium above in the absence of GCs.

For studying human oogenesis *in vitro*, the cells were trypsinized and 2 × 10^4^ cells were cultured on a GC monolayer per well of a 24-well plate. At the first stage, the cells were cultured in basic medium containing 2 μM RA for 3 days. At the second stage, the cells were cultured in basic medium containing 10 ng/mL human EGF, 5 μg/mL transferrin (Sigma), 10 μg/mL insulin (Sigma), and 1 U/mL of each gonadotropin (hCG and PMSG, Prospect) for 6 days. Finally, the cells cultured in the medium at the second stage were supplied with 1 ng/mL progestogen (Sigma), 1 ng/mL 17-β-estradiol (E2, Sigma), and 10% human follicular fluid (HFF, it was centrifuged for 30 min at 300 g and heat inactivated at 56 °C for 30 min. Then, it was aliquoted and stored at −20 °C until use) for 3–6 days. The medium was changed daily. All cultures were maintained at 37 °C in a 5% CO_2_-95% air atmosphere.

### Gap-junctional communication assay

To determine the function of GJIC between the OLCs and GCs *in vitro*, a GC monolayer was preloaded with calcein AM (3′,6′-Di(O-acetyl)-2′,7′-bis[N,N-bis(carboxymethyl) amino methyl]-fluorescein, tetraacetoxy methyl ester, YEASEN, 40719ES50) according to the manufacturer’s instructions. Subsequently, 2 × 10^4^ of faFGSCs were seeded onto the GC monolayer. Calcein transfer from preloaded GCs to unlabeled OLCs was determined by fluorescence microscopy 24 h later.

### Meiotic spreads and SYCP3 staining

Meiotic spreads of cell samples were prepared as described previously^53^. For SYCP3 and γH2AX staining, the slides were incubated for 10 min at 37 °C with blocking buffer (PBS containing 10% normal goat serum) and subsequently incubated overnight with a 1:200 dilution of rabbit polyclonal SYCP3 (Abcam; ab150292) and a 1:200 dilution of mouse monoclonal γH2AX (Abcam; ab22551) in a humidified chamber at 4 °C. After washing in PBS, the slides were incubated at 37 °C for 30 min with a 1:150 dilution of TRITC conjugated secondary antibody (goat anti-rabbit IgG) and a 1:150 dilution of FITC conjugated secondary antibody (goat anti-mouse IgG), then incubated at 37 °C for 20 min with 500 ng/mL of DAPI and mounted as described above. Images were obtained with a Leica DM2500 microscope and a Leica DFC 550 digital camera.

### RT-PCR and qRT-PCR

Total RNA exaction from cells and tissues was performed as previously described^11^. Reverse transcription was performed using a HiScript^®^ II Q RT SuperMix (+gDNA wiper) kit (Vazyme, R223-01), according to the manufacturer’s instructions. For RT-PCR, 35 PCR cycles were performed using Taq polymerase (Takara, R10T1M) with primer sets specific for each gene (Supplementary Table S2). The glyceraldehyde-3-phosphate dehydrogenase (*GAPDH*) gene was amplified in each sample as a loading control. Samples were resolved through 2% agarose gels and run under the same experimental conditions. DNA bands were detected using EB (ethidium bromide) staining. PCR products were isolated, sub-cloned, and sequenced to confirm the gene sequences. For qRT-PCR, the specificity of PCRs was verified by a single peak according to melt curves. qRT-PCR was performed with a 7500 real-time PCR amplification system using SYBR Green PCR master mix (Roche). The relative levels of transcripts were calculated using the ΔΔCT method within the ABI 7500 System Software (V2.0.4) and all of the gene expression levels were normalized to *GAPDH*.

For analysis DDX4 sorted cells and single OLC by RT-PCR, 5–10 cells associated with the magnetic beads after MACS with DDX4 antibody or OLCs in the medium were picked under an inverted microscope using a mouth pipette and transferred into RNase-free PBS. Subsequently, OLCs were transferred into 5.5 μL of lysis mix (1 × PCR buffer [Takara, 9151A] and 0.4 U/μL rRNasin [Takara, 2313A]). PCR tubes containing lysed cells were heated to 65 °C for 10 min and cooled at 4 °C. Reverse transcription was performed using the HiScript^®^ II Q RT SuperMix (+gDNA wiper) kit, according to the manufacturer’s instructions. RT-PCR analysis of DDX4 sorted cells was performed as described previously^54^. For analysis single OLC, 45 PCR cycles were performed using Taq polymerase with primer sets specific for each gene. Samples were resolved through 2% agarose gels and DNA bands were detected using EB staining. PCR products were isolated, sub-cloned, and sequenced to confirm the gene sequences.

### Time-lapse analysis

Time-lapse analysis was performed using an inverted Olympus IX81 Microscope (Olympus, Tokyo, Japan) controlled by Xcellence software. During imaging, cells in a 6-well culture-plate were enclosed in a chamber maintained at 37 °C under a humidified atmosphere of 5% CO_2_-95% air atmosphere. Time-lapse images were taking one frame every 10 min or 15 min over 24 h of culture using a 4× phase contrast objective.

### Statistics

All data were expressed as mean ± SEM. Statistical tests were carried out using a student’s t-test or one-way analysis of variance followed by the Tukey’s *post hoc* test with Statistical Package for the Social Sciences (SPSS) software (version 20.0; IBM). Values with P < 0.05 were considered statistically significant, and values with P < 0.01 were considered extremely significant. Graph generation was performed using SigmaPlot (version 13.0; www.sigmaplot.com) or R Project software (version 3.1.3; www. R-project.org).

## Additional Information

**How to cite this article**: Ding, X. *et al*. Human GV oocytes generated by mitotically active germ cells obtained from follicular aspirates. *Sci. Rep.* **6**, 28218; doi: 10.1038/srep28218 (2016).

## Supplementary Material

Supplementary Information

Supplementary Figure S5

Supplementary Video 1

Supplementary Video 2

## Figures and Tables

**Table 1:** Adult human ovarian samples surgically removed from different patients used for gene expression analysis, FGSC isolation and transplantation.

Patients	Age	Pathology	Procedure of Sample Taken	Applications
A	28	Ovarian Cyst	Laparoscopy	RT-PCR
B	43	Cervical Carcinoma	Fully Oophorectomy	FGSC Isolation
C	28	Ectopic Pregnancy	Laparoscopy	FGSC Isolation
D	26	Ovarian Cyst	Laparoscopy	FGSC Isolation
E	26	Ovarian Cyst	Laparoscopy	Transplantation
F	31	Ovarian Cyst	Laparoscopy	Transplantation
G	28	Ovarian Cyst	Laparoscopy	Transplantation
