# Supplementary material for: Human GV oocytes generated by mitotically active germ cells obtained from follicular aspirates
Source: Sci Rep. 2016 Jun 30;6:28218. doi: 10.1038/srep28218 (PMC4928061; doi:10.1038/srep28218)
Supplement: Supplementary Information [file srep28218-s1.pdf]

Human GV oocytes generated by mitotically active germ cells obtained from follicular aspirates

Xinbao Ding[1], Guishu Liu[3], Bo Xu[1], Changqing Wu[1], Ning Hui[6], Xin Ni[7], Meirong Du[5*], Xiaoming Teng[4*], Ji Wu[1, 2, 8*]

[1] Renji Hospital Shanghai Jiaotong University School of Medicine, Key Laboratory for the Genetics of Developmental & Neuropsychiatric Disorders (Ministry of Education), Bio-X Institutes, Shanghai Jiao Tong University, Shanghai 200240, China

[2] Key Laboratory of Fertility Preservation and Maintenance of Ministry of Education, Ningxia Medical University, Yinchuan 750004, China

[3]The First People's Hospital of Chenzhou, Chenzhou 42300, Hunan, China

[4]Center of Reproductive medicine, Shanghai First Maternity and Infant Hospital, Tongji University School of Medicine, Shanghai 200040, China

[5] Laboratory for Reproductive Immunology, Obstetrics and Gynecology Hospital, Fudan University, Shanghai 200011, China; Shanghai Key Laboratory of Female Reproductive Endocrine Related Diseases, Shanghai, 200011, China

[6] Changhai Hospital of Second Military Medical University, Shanghai 200433, China

[7]Department of Physiology, Second Military Medical University, 800 Xiangyin Road, Shanghai 200433, China

[8]Shanghai Key Laboratory of Reproductive Medicine, Shanghai 200025, China

Correspondence to: Ji Wu, Bio-X Institutes, Shanghai Jiao Tong University, No. 800. DongchuanRoad, Minhang District, Shanghai, 200240, China.

Phone: 86-21-34207263; Fax: 86-21-34204051; E-mail: jiwu@sjtu.edu.cn; or to Xiaoming Teng, e-mail: tengxiaoming@hotmail.com; or Meirong Du, e-mail: mrdu@fudan.edu.cn

Supplementary Figures

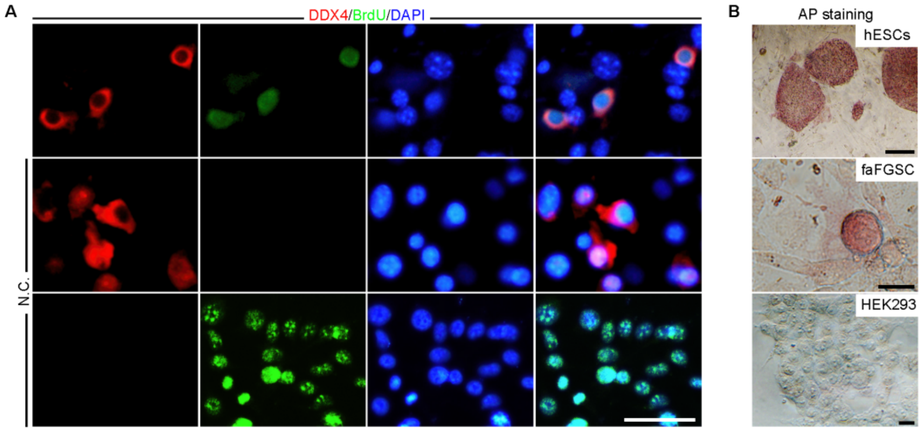

Figure S1. Dual immunofluorescence analysis of DDX4 and BrdU, and alkaline phosphatase staining for faFGSCs.

(**A**) Dual immunofluorescence analysis of faFGSCs for DDX4 (red) and BrdU (green). The nuclei are counterstained with DAPI. N.C. is the omission of the primary antibody. (**B**) Alkaline phosphatase (AP) staining of human ESCs, faFGSCs, and HEK293 cells. Scale bars: 10 μm (middle panel of **B**), 50 μm (**A** and lower panel of **B**), 100 μm (upper panel of **B**).

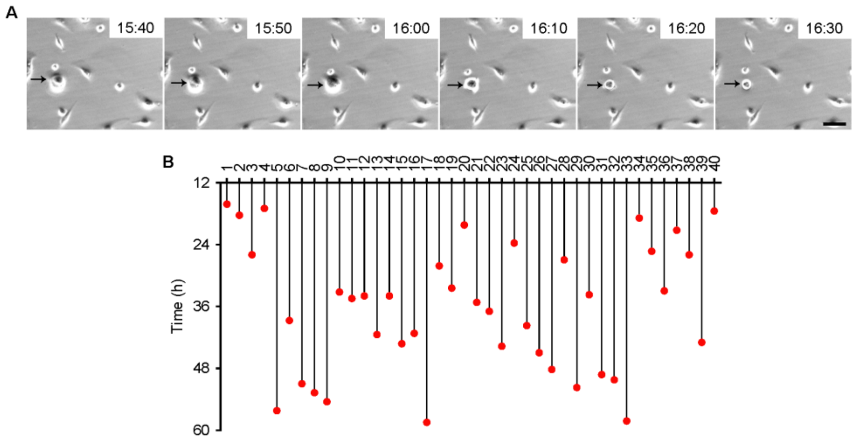

Figure S2. Time-lapse analysis for faFGSC spontaneous differentiation *in vitro*.

(A) Phase contrast images obtained by video time-lapse microscopy at different time points (hour:minute) show a suspended OLC (black arrow) morphologically transformed from adherent faFGSC when cultured under feeder-free conditions (see also **Video S1** in the supplementary materials). (B) Cell fate of faFGSCs from 12 h to 60 h when cultured under feeder-free conditions (n=40). Red cycles indicate the cell morphologically transformed to suspended OLCs. Scale bars: 100 μm (**A**).

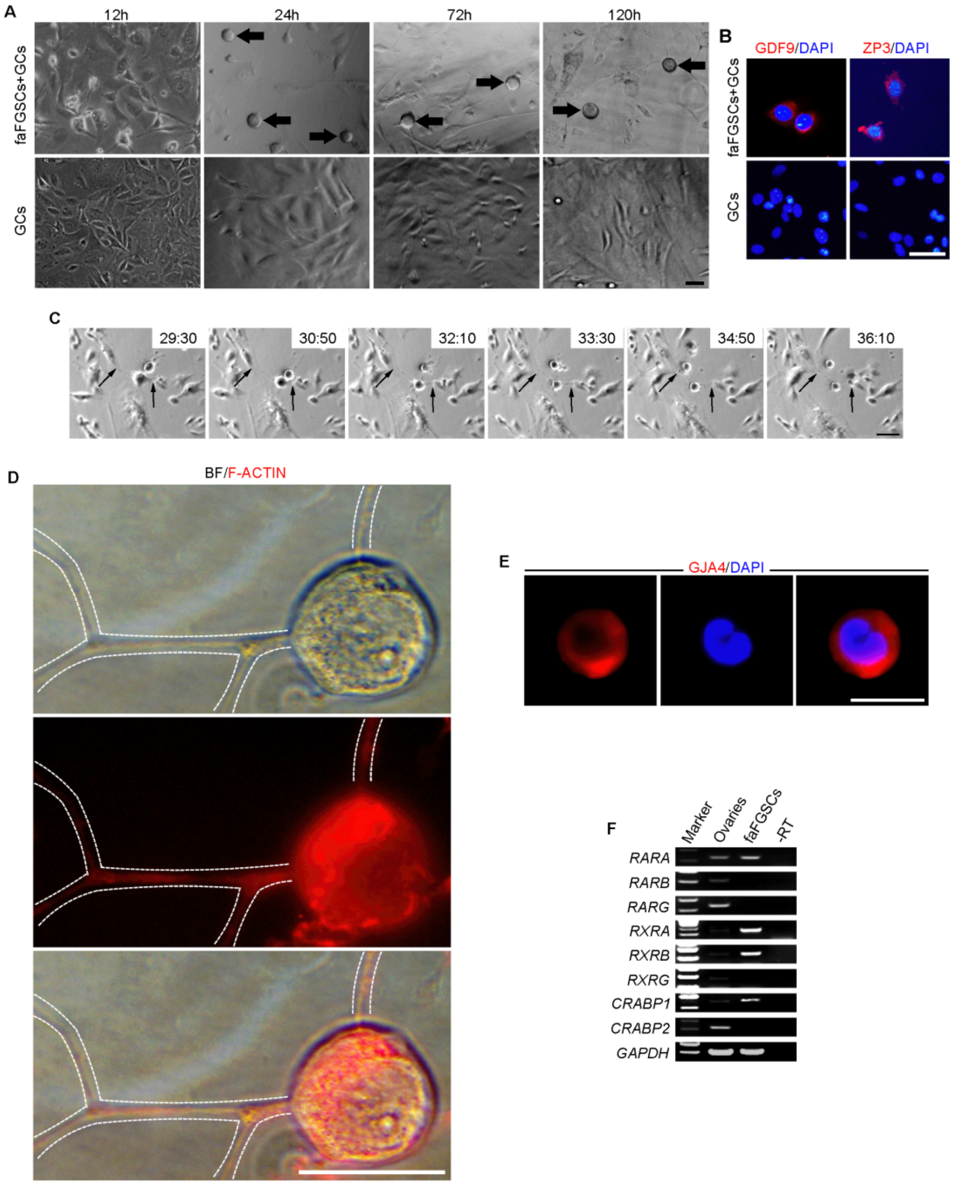

Figure S3. The development of OLCs from faFGSCs were regulated by retinoic acid and granulosa cells.

(**A**) Representative morphologies of female germ cells from faFGSCs cultured on GC monolayer (faFGSCs + GCs, upper panel) and GCs alone (GCs, lower panel) after RA treatment at 12 h, 24 h, 72 h, and 120 h, respectively. (**B**) Immunofluorescence analysis of GDF9 and ZP3 for faFGSCs cultured under a RA-supplemented and GC monolayer at 72 h. Immunofluorescence analysis of GDF9 and ZP3 in GCs served as

negative controls. (**C**) Phase contrast images obtained by video time-lapse microscopy at different time points (hour:minute) show OLCs' morphological transformation from faFGSCs when cultured with GCs and RA treatment. Black arrows indicate the tentacle-like structures between an OLC with surrounding GCs (see also **Video S2** in the supplementary materials). (**D**) Immunofluorescence analysis of tentacle-like structures (marked by dotted line) with F-ACTIN (middle panel). (**E**) Immunofluorescence analysis of OLC for GJA4. (**F**) Expression analysis of mRNAs encoding candidate RA receptors (*RARA*, *RARB*, and *RARG*), retinoid X receptors (*RXRA*, *RXRB*, and *RXRG*) and RA binding proteins (*CRABP1* and *CRABP2*) in human ovaries (28 years old) and faFGSCs by RT-PCR. The nuclei in **B** and **E** are counterstained with DAPI. Scale bars: 50 μm (**A**, **B**, **D**, and, **E**), 100 μm (**C**).

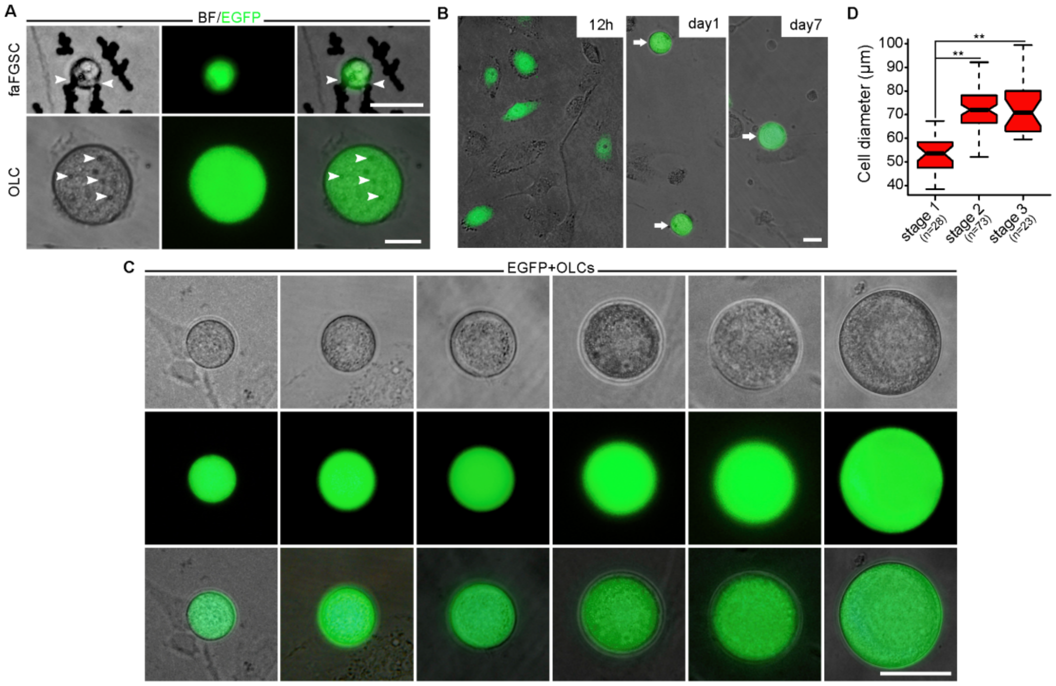

Figure S4. Development of OLCs from EGFP-labeled human faFGSCs.

(**A**) Representative bright-field (BF, left panel), EGFP (middle panel), and merged (right panel) images of faFGSC (upper panel) and OLC (lower panel). The white arrowheads indicate the magnetic beads. (**B**) Representative morphologies (merged by bright-field and EGFP, indicated by white arrows) of female germ cells from faFGSCs during *in vitro* differentiation at 12 h, day 1, and day 7. (**C**) Representative morphologies of EGFP$^+$ OLCs observed during differentiation. (**D**) Boxplots showing the distribution of the diameter of EGFP$^+$ OLCs formed by faFGSCs at stage 1 (52.9 ± 1.3 µm; mean ± SEM), stage 2 (72.1 ± 1 µm), and stage 3 (73.6 ± 2.6 µm). Numbers in brackets denote the number of OLCs measured. Black central line represents the median, notch represents 95% median confidence intervals, boxes and whiskers represent the 25th and 75th, and 2.5th and 97.5th percentiles. Data were

analyzed using one-way analysis of variance followed by the Tukey's post hoc test. **indicates $P < 0.01$. Scale bars: 25 µm (**A**), 50 µm (**B** and **C**).

Supplementary Tables

Table S1. No. of ART cycles and babies born during 2000-2007 reported by International Committee for Monitoring Assisted Reproductive Technologies (ICMART).

Year	No. of ART cycles	No. of babies born	No. of reporting clinics	No. of reporting countries and regions	Reference
2000	460,157	197,000-220,000	1,429	49	[1]
2002	601,243	219,000-246,000	1,563	53	[2]
2003	433,427	173,424	1,709	54	[3]
2004	954,743	237,809	2,184	52	[4]
2005	1,052,363	237,315	2,973	53	[5]
2006	1,050,300	256,668	2,352	56	[6]
2007	1,251,881	229,442	2,419	55	[7]

Table S2. RT–PCR and qRT-PCR (labeled by *) primers for gene expression analysis.

Gene	Accession number	Product Size (bp)	Primer Sequence (5' - 3')[#]
DDX4	NM_024415	139	F:CGTTGAAATTCTGCGAAACA<br>R:TCTCTGTTCCCGATCACCAT
IFITM3	NM_021034	214	F:ACCATGTCGTCTGGTCCCTGT<br>R:AGCACTGGGATGACGATGAGCAGA
OCT4	NM_002701	564	F:TGAGGGCGAAGCAGGAGT<br>R:TGGCGCCGGTTACAGAA
STELLA	NM_199286	168	F:GTTACTGGGCGGAGTTCGTA<br>R:TGAAGTGGCTTGGTGTCTTG
DAZL	NM_001190811	163	F:GCCCACAACCACGATGAATC<br>R:CGGAGGTACAACATAGCTCCTTT
BLIMP-1	NM_001198	300	F:GGGTGCAGCCTTTATGAGTC<br>R:CCTTGTTCATGCCCTGAGAT
*STRA8**	NM_182489	220	F:ACTCTCAGTCTGATCTCATAGCC<br>R:TACCAAGGGGAGGAACCATTC
*SYCP3**	NM_001177949	136	F:TCAGAGCCAGAGATTGAAAACA<br>R:TTGCAACATAGCCATTTCTTTTT
C-KIT	NM_000222	291	F:GGCATGCTCCAATGTGTGG<br>R:GGTGTGGGGATGGATTTGC
FIGLA	NM_001004311	155	F:GGCAAGACAGCTGTCAAGA<br>R:TTGGGGAGATAATTTCAGTCGT
*FIGLA**	NM_001004311	214	F:ATCTCAACCGTGGTTTTGCCA<br>R:TGTCTTGCCGAGGATGTATGT
*GDF9**	NM_005260	118	F:ATGGCACGTCCCAACAAATTC<br>R:ACTCAGCACTAGCAGCAATCT
*GJA4**	NM_002060	119	F:TGCAAGAGTGTGCTAGAGGC<br>R:ACAAAGCAGTCCACGAGGTAG
ZP1	NM_207341	219	F:CGCCATGTTCTCTGTCTCAA

			R:CGTTTGTTCACATCCCAGTG
*ZP1**	NM_207341	131	F:ACGACCTGGGGTTACCCTG<br>R:AGCTGCATTCCCTTGATCCC
ZP2	NM_003460	245	F:GCCTCCCAGGACCCATTCTC<br>R:CAGGTAGCAGATGGAGCCTA
ZP3	NM_001110354	273	F:AGCAGGACCCAGATGAACTCAACA<br>R:AAGCCCACTGCTCTACTTCATGGT
*ZP3**	NM_001110354	116	F:ACTCCACGAGTGTGGCAAC<br>R:CGCGGTTAGTCCTCACGAT
NANOG	NM_024865	215	F:CAGAAGGCCTCAGCACCTAC<br>R:CTGTTCCAGGCCTGATTGTT
SOX-2	NM_003106	437	F:ATGCACCGCTACGACGTGA<br>R:CTTTTGCACCCCTCCCATTT
REX-1	NM_174900	306	F:CAGATCCTAAACAGCTCGCAGAAT<br>R:GCGTACGCAAATTAAAGTCCAGA
*LHX8**	NM_001001933	93	F:AGCTTCAGGTTATGCAAGCAC<br>R:GTCTGCTCAAGCCTGTCCTTT
*NOBOX**	NM_001080413	99	F:GAGACCCTCAAATCACCCCAA<br>R:GCCCCTTGTGAGTTCCCTTTT
*CDKN1A**	NM_078467	220	F:CGATGGAACTTCGACTTTGTCA<br>R:GCACAAGGGTACAAGACAGTG
*DMC1**	NM_007068	185	F:AGAAACATGGAATTAACGTGGCT<br>R:AAATGCAGTCAAGAATCCTGGTT
*SPO11**	NM_012444	219	F:ACAGAGCAACACTTATGCAACC<br>R:GCACCACAGGTACAATTCACT
*MLH1**	NM_000249	76	F:CTCTTCATCAACCATCGTCTGG<br>R:GCAAATAGGCTGCATACACTGTT
*MSH5**	NM_025259	91	F:AGTGACTCCACTATCCACTTCAT<br>R:ACTGGGGATTGATCTCATCCA

*PRDM9**	NM_020227	233	F:CAGCCAACAATGGATACTCCTG<br>R:CTGGCCGTATTCATCCCCA
RARA	NM_000964	122	F:AAGCCCGAGTGCTCTGAGA<br>R:TTCGTAGTGTATTTGCCCAGC
RARB	NM_000965	209	F:CCCCAGAACAAGACACCATGA<br>R:TTTTGTCGGTTCCTCAAGGTC
RARG	NM_000966	234	F:TGTCACCGCGACAAAAACTGT<br>R:CGAGGGGAAAGTCTCCTGA
RXRA	NM_002957	246	F:GGACTGCCTGATTGACAAGC<br>R:TTCAGCCCCATGTTTGCCTC
RXRB	NM_001270401	234	F:TTGCCGGGACAACAAAGACT<br>R:CCCTGGTCACTCTTCTGTTCC
RXRG	NM_006917	185	F:AGGGAAGCTGTGCAAGAAGAA<br>R:GTAACAGGGTCATTTGTCGAGT
CRABP1	NM_004378	143	F:GCAGCAGCGAGAATTTCGAC<br>R:CGTGGTGGATGTCTTGATGTAGA
CRABP2	NM_001878	219	F:ATCGGAAAACTTCGAGGAATTGC<br>R:AGGCTCTTACAGGGCCTCC
GAPDH	NM_002046	210	F:GACATCAAGAAGGTGGTGAAGC<br>R:GTCCACCACCCTGTTGCTGTAG
*GAPDH**	NM_002046	197	F:GGAGCGAGATCCCTCCAAAAT<br>R:GGCTGTTGTCATACTTCTCATGG

#, F: forward primer; R: reverse primer.

Supplementary Videos

Video S1. Time-lapse video of female germ cell (black arrow) from faFGSC cultured under a feeder-free condition at low density between 12 h and 23 h. Scale bar: 100 μm.

Video S2. Time-lapse video of female germ cells (black arrow) from faFGSCs co-cultured with GCs between 19 h and 37 h. Female germ cells built tentacle-like structures with surrounding GCs are shown. Scale bar: 100 μm.

References

1 Adamson, G. D. *et al.* World collaborative report on in vitro fertilization, 2000. *Fertil Steril* **85**, 1586-1622, (2006).

2 de Mouzon, J. *et al.* World Collaborative Report on Assisted Reproductive Technology, 2002. *Hum Reprod* **24**, 2310-2320, (2009).

3 Nygren, K. G. *et al.* International Committee for Monitoring Assisted Reproductive Technology (ICMART) world report: assisted reproductive technology 2003. *Fertil Steril* **95**, 2209-2222.e2217, (2011).

4 Sullivan, E. A. *et al.* International Committee for Monitoring Assisted Reproductive Technologies (ICMART) world report: assisted reproductive technology 2004. *Hum Reprod* **28**, 1375-1390, (2013).

5 Zegers-Hochschild, F. *et al.* International Committee for Monitoring Assisted Reproductive Technology: world report on assisted reproductive technology,

2005. *Fertil Steril* **101**, 366-378.e314, (2014).

6 Mansour, R. *et al.* International Committee for Monitoring Assisted Reproductive Technologies world report: Assisted Reproductive Technology 2006. *Hum Reprod* **29**, 1536-1551, (2014).

7 Ishihara, O. *et al.* International Committee for Monitoring Assisted Reproductive Technologies: World Report on Assisted Reproductive Technologies, 2007. *Fertil Steril* **103**, 402-413.e411, (2015).
