# Supplementary material for: Human GV oocytes generated by mitotically active germ cells obtained from follicular aspirates
Source: Sci Rep. 2016 Jun 30;6:28218. doi: 10.1038/srep28218 (PMC4928061; doi:10.1038/srep28218)
Supplement: Supplementary Figure S5 [file srep28218-s2.doc]

**Human GV oocytes generated by mitotically active germ cells obtained from follicular aspirates**

Xinbao Ding^1^, Guishu Liu^3^, Bo Xu^1^, Changqing Wu^1^, Ning Hui^6^，Xin Ni^7^，Meirong Du^5*^，Xiaoming Teng^4*^，Ji Wu^1, 2, 8*^

^1^ Renji Hospital Shanghai Jiaotong University School of Medicine, Key Laboratory for the Genetics of Developmental & Neuropsychiatric Disorders (Ministry of Education), Bio-X Institutes, Shanghai Jiao Tong University, Shanghai 200240, China

^2^ Key Laboratory of Fertility Preservation and Maintenance of Ministry of Education, Ningxia Medical University, Yinchuan 750004, China

^3^The First People’s Hospital of Chenzhou, Chenzhou 42300, Hunan, China

^4^Center of Reproductive medicine, Shanghai First Maternity and Infant Hospital, Tongji University School of Medicine, Shanghai 200040, China

^5^ Laboratory for Reproductive Immunology, Obstetrics and Gynecology Hospital, Fudan University, Shanghai 200011, China; Shanghai Key Laboratory of Female Reproductive Endocrine Related Diseases, Shanghai, 200011, China

^6^ Changhai Hospital of Second Military Medical University, Shanghai 200433, China

^7^Department of Physiology, Second Military Medical University, 800 Xiangyin Road, Shanghai 200433, China

^8^Shanghai Key Laboratory of Reproductive Medicine, Shanghai 200025, China

Correspondence to: Ji Wu, Bio-X Institutes, Shanghai Jiao Tong University, No. 800. DongchuanRoad, Minhang District, Shanghai, 200240, China.

Phone: 86-21-34207263; Fax: 86-21-34204051; E-mail: jiwu@sjtu.edu.cn; or to Xiaoming Teng, e-mail: tengxiaoming@hotmail.com; or Meirong Du, e-mail: mrdu@fudan.edu.cn


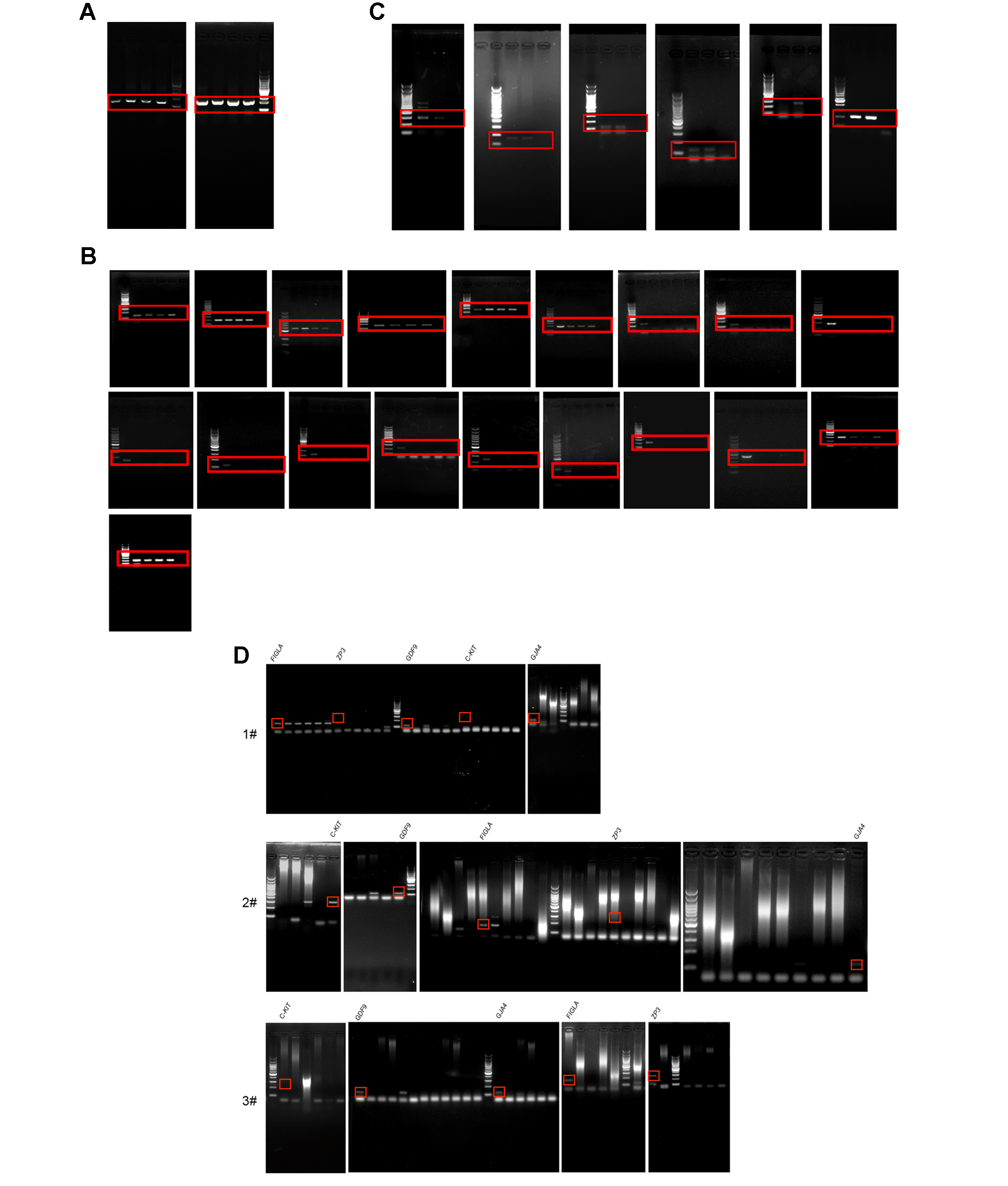


**Figure S5.** **Uncropped gels.**

(**A**) Uncropped gels for Figure 1E. (**B**) Uncropped gels for Figure 3F. (**C**) Uncropped gels for Figure 4F. (**D**) Uncropped gels for Figure 4G. Red boxes correspond to those shown in the cropped images within the manuscript.
